# Supplementary material for: The regenerative role of neural crest stem cells in physical stimuli-enhanced peripheral nerve repair
Source: Stem Cell Reports. 2026 Mar 26;21(4):102861. doi: 10.1016/j.stemcr.2026.102861 (PMC13083801; doi:10.1016/j.stemcr.2026.102861)
Supplement: Document S1. Figures S1–S6, Table S1, and supplemental methods [file mmc1.pdf]

Stem Cell Reports, Volume *21*

Supplemental Information

The regenerative role of neural crest stem cells in physical stimuli-enhanced peripheral nerve repair

Youyi Tai, Lu Jin, Thamidul Islam Tonmoy, B. Hyle Park, and Jin Nam

Supplemental figures

A

B

C

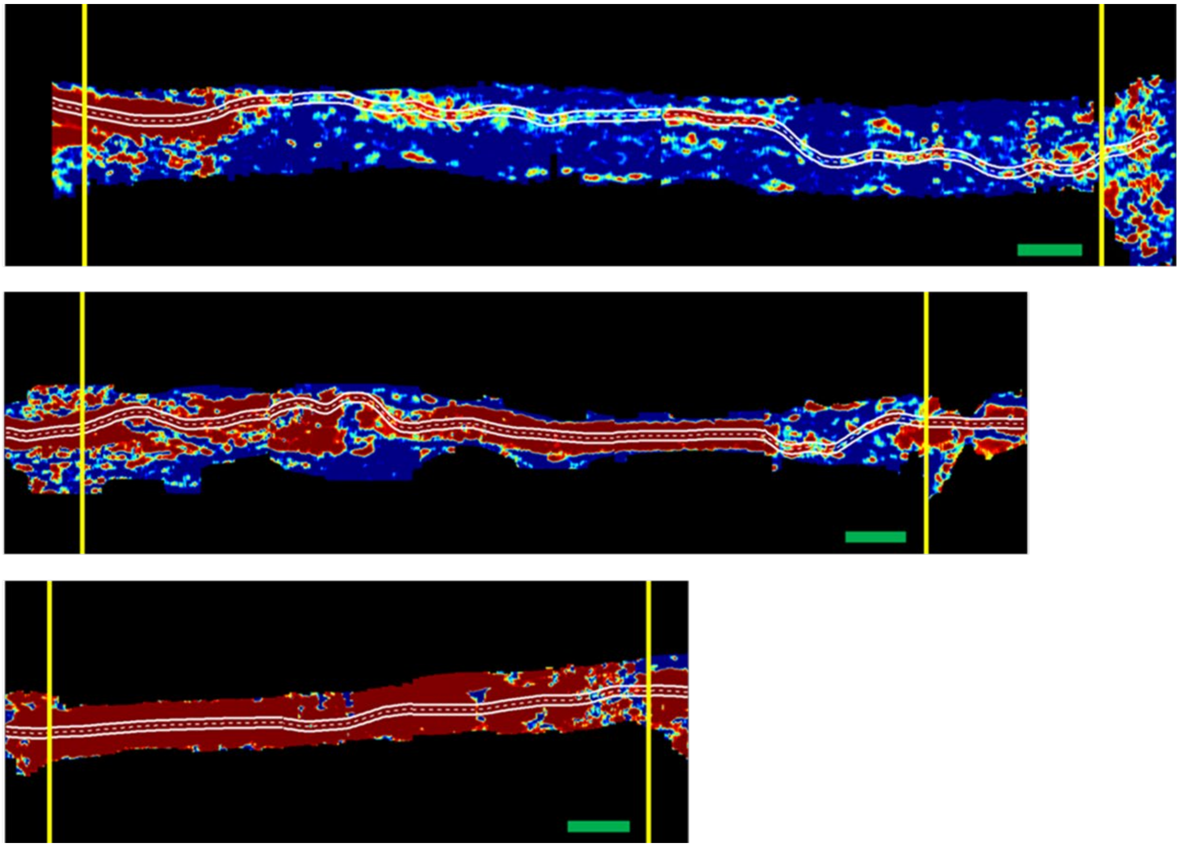

Figure S1. Representative enface phase-retardation images and manually traced optimal nerve connection paths. (A), (B) and (C) are the enface phase retardation images of the whole length of a static, MES, and healthy control sample, respectively. In these images, the color scale from blue to red represents increasing phase retardation within the range of 0.07 to 0.2 deg/μm. The left side corresponds to the proximal end of the nerve. Vertical yellow lines mark the proximal and distal boundaries of the conduit. Dashed white lines indicate the manually traced optimal nerve connection paths, while the solid white lines delineate the 150 μm-wide regions used for averaging. Scale bar = 1mm.

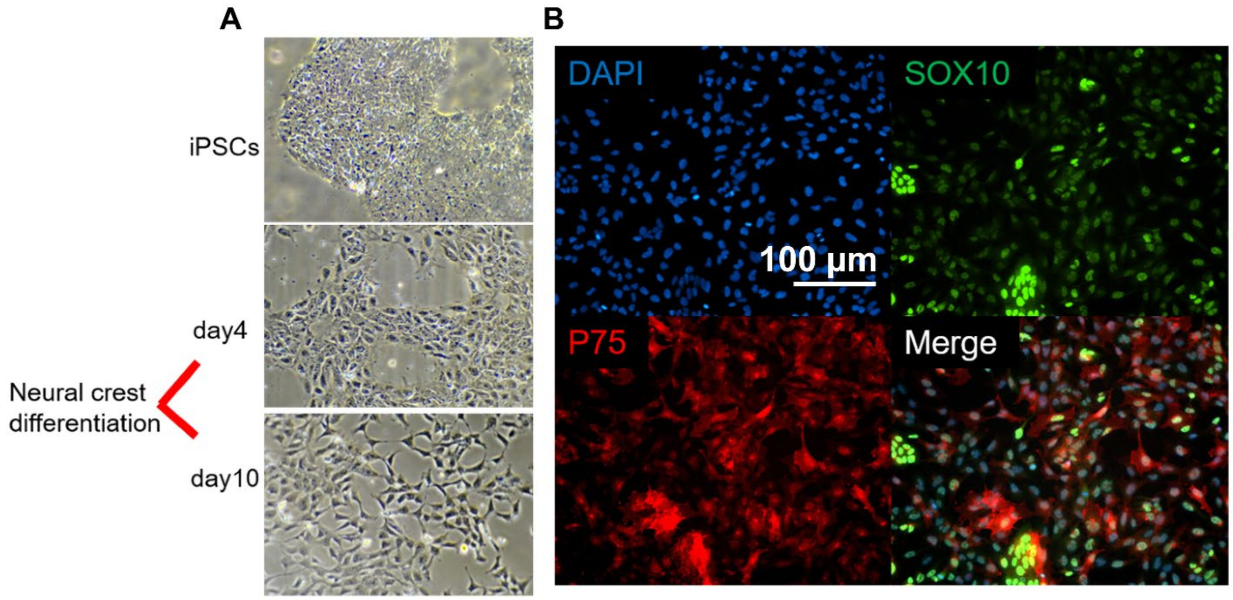

Figure S2. Derivation and characterization of neural crest stem cells (NCSCs)-like cells from induced pluripotent stem cells (iPSCs). (A) Bright-field images showing the morphology of iPSCs and NCSC-like cells. (B) Immunofluorescent images of neural crest markers SOX10 and P75NTR, counter-stained with DAPI.

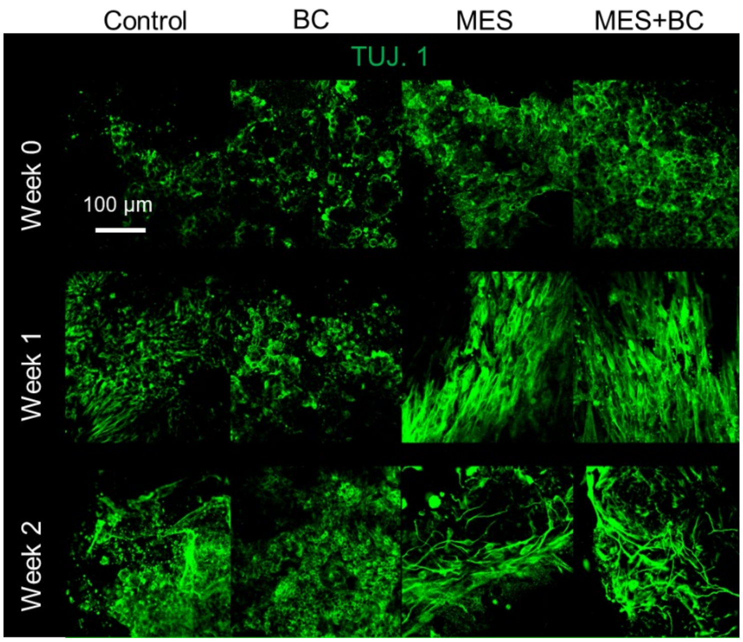

Figure S3. Green channel of fluorescence images (TUJ. 1) in Figure 2B.

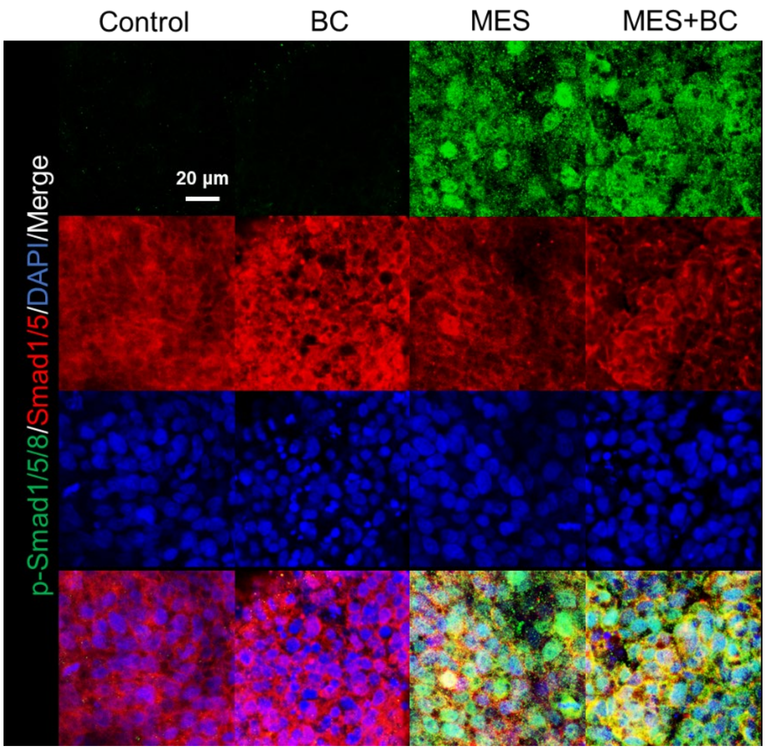

Figure S4. Effect of physical/biochemical stimulation on the expression of Smad. Confocal images showing the expression of p-Smad1/5/8 (green) and Smad1/5 (red) after the first stimulation at week 0 under the Control, Biochemical stimulation (BC), Mechano-electrical stimulation (MES), and MES+BC conditions. The cells were stimulated for 2 hours before sample fixation.

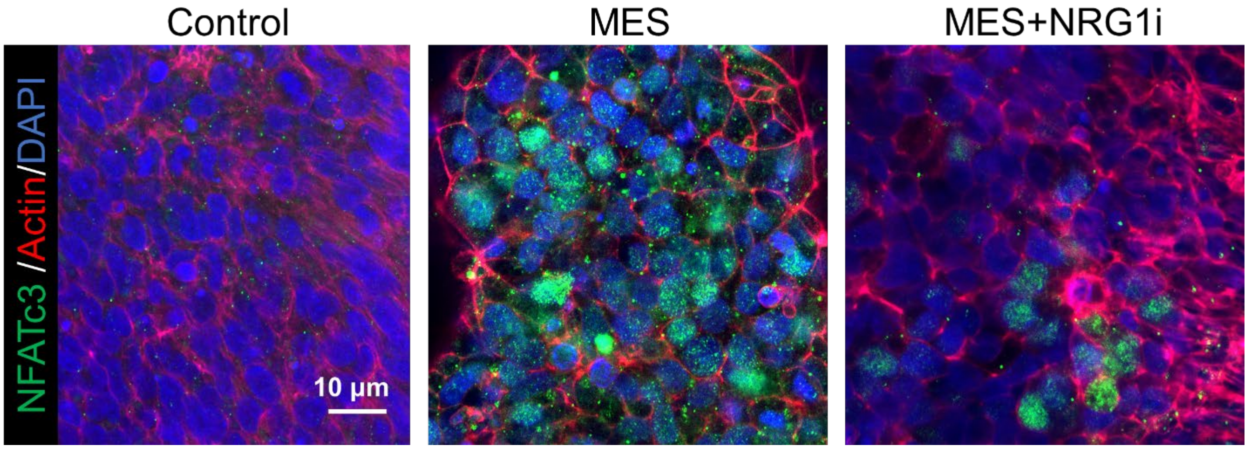

Figure S5. **Effect of NRG1 inhibitor on NFATc3 expression under Control, mechano-electrical stimulation (MES), and MES+NRG1 inhibitor (MES+NRG1i) conditions.**

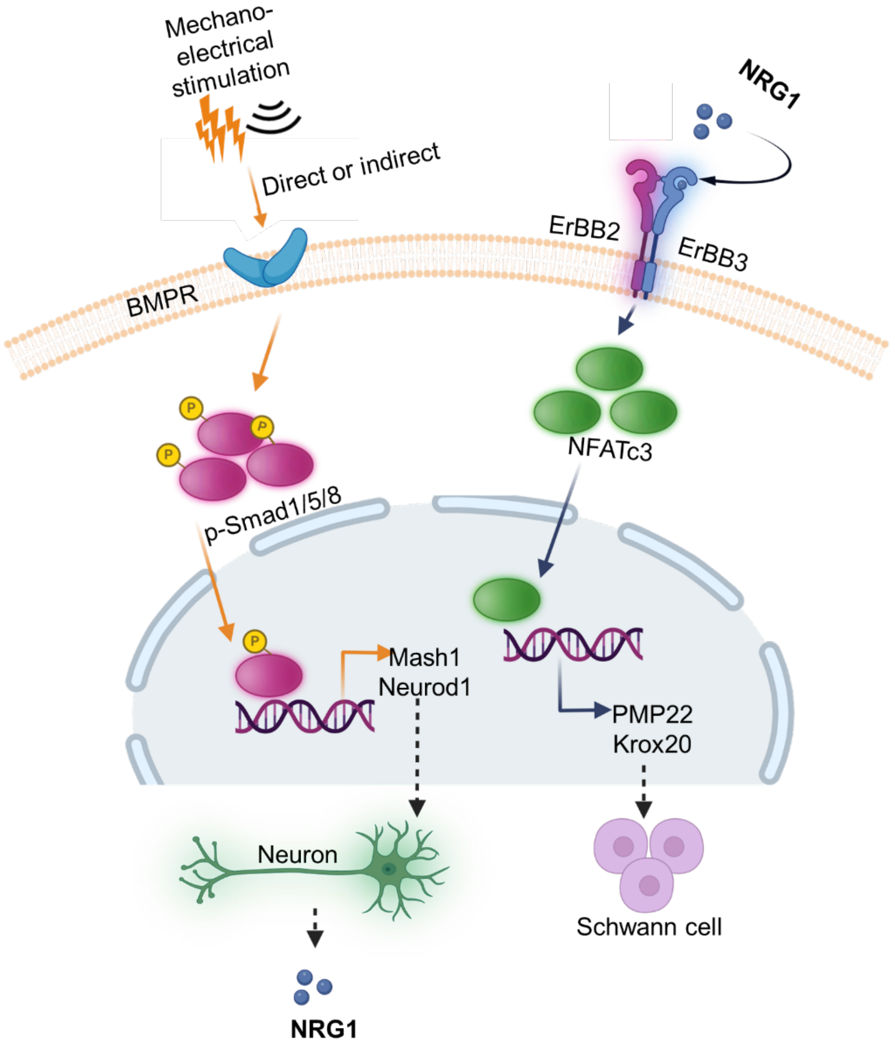

Figure S6. Schematic of the signaling mechanism that mediates the MES-induced NCSC-like cells differentiation towards neurons and Schwann cells.

Supplemental table

Table S1 (refer to Figures 2-4). Human primers for the qRT-PCR analysis.

Primer	Forward	Reverse
GAPDH	5’-ATGGGGAAGGTGAAGGTCG-3’	5’-TAAAAGCAGCCCTGGTGACC-3’
NEUROD1	5’-AAAGCCCTCTGACTGATTGCA-3’	5’-GGACGGTTCGTGTTTGAAAGA-3’
MASH1	5’-ACTTGAACTCCATGGCCGGCT-3’	5’-CCAGTTGGTGAAGTCGAGAAG-3’
NGN2	5’-CATCAAGAAGACCCGTAGACTGA-3’	5’-TCTCGATCTTGGTGAGCTTGG-3’
KROX20	5’-TGCACCTAGAAACCAGACCTT-3’	5’-ATGCCTGCACTCACAATATTG-3’
NCAM1	5’-TGGAATGCTGAGTATGAGGTC-3’	5’-TGAACACAAAATGAGCCGCCT-3’
PMP22	5’-TGTAGCACCTCTTCCTCAG-3’	5’-GAGTTGGCAGAAGAACAGGAAC-3’

Supplemental methods

Synthesis of electrospun poly(vinylidenefluoride-trifluoroethylene)(P(VDF-TrFE)) scaffolds

P(VDF-TrFE) fibers having an average fiber diameter of 500 nm were synthesized using an electrospinning technique as described previously [S1]. Briefly, a solution of 7 wt.% P(VDF-TrFE) (70:30 mol%, Solvay) dissolved in a solvent mixture of N,N-dimethylformamide (DMF) (Sigma) and acetone (Fisher) in a 60:40 volume ratio, supplemented with 1.5 wt.% pyridinium formate (PF) buffer (Sigma), was prepared. Electrospinning was performed under optimized conditions, including a needle tip-to-collector distance of ~10 cm, an applied voltage of −15 to −20 kV, a solution feed rate of 6 mL/h, and an absolute humidity of 7.6 g m^{-3} at room temperature (23 °C). A rotating wheel with an angular velocity of 47.9 m s^{-1} was used for fiber collection, resulting in aligned fibrous scaffolds. The process duration was adjusted to produce scaffolds with a thickness of approximately 200 µm. To enhance piezoelectric properties, the scaffolds were annealed at 90°C for 24 hours [S1].

Surgical procedures and nerve-conduit harvesting

Animal experiments were conducted as previously described [S2] and approved by the University of California-Riverside (UCR) Institutional Animal Care and Use Committee (IACUC, protocol 20210016), in compliance with ethical regulations. Adult Sprague Dawley rats (n = 9, Taconic) weighing ~300 g were randomly divided into three groups. Rats underwent sciatic nerve transection surgery, and the severed nerve ends were sutured to P(VDF-TrFE) conduits, forming a 15 mm nerve gap. Experimental rats (MES group, n = 3) received periodic shockwave treatments (twice a week) post-surgery, while control rats (Static group, n = 3) did not receive mechano-electrical stimulation. No sciatic nerve transection or conduit transplantation was performed on Healthy control rats (n = 3). To apply the mechano-electrical stimulation, a therapeutic shockwave system was used to activate the piezoelectric P(VDF-TrFE) conduits bridging the transected nerves, generating 3 Hz of pulsed biphasic electric signals with 200 $mV_{p\text{-}p}$. At 12 weeks post-surgery, rats were euthanized. Implanted conduits were excised and subjected to further analyses. All samples were fixed in 2% glutaraldehyde/2% paraformaldehyde solution at 4 °C for 48 hours before OCT imaging and histological analysis.

Polarization-sensitive optical coherence tomography (PS-OCT) imaging

Fixed samples were imaged with a custom-built spectral domain polarization-sensitive optical coherence tomography (PS-OCT) system with a central wavelength of 1310 nm and a full-width at half-maximum (FWHM) bandwidth of 68 nm. The axial and lateral resolutions of the system are 11 µm and 37 µm, respectively, and an imaging depth of 2 mm. A comprehensive description of the hardware, scanning, and data processing pipeline has been reported previously [S2]. In brief, each sample was scanned volumetrically in a series of overlapping sections to capture the full length, with each section covering a 4.5 mm × 4.5 mm lateral field of view and an overlap of approximately 1 mm between adjacent sections to ensure proper registration and alignment. The sections were manually aligned when generating the full length of the sample. From volumetric data, structural intensity images were generated following the standard Fourier domain processing method [S3]. The surface of the nerve within the conduit was manually delineated in intensity images and served as the polarization reference for computing cumulative phase-retardation images with a Stokes vector approach [S4]. The slope of the rising portion of the cumulative phase retardation curve, which is proportional to birefringence [S5], was measured using a linear least square fit to generate enface phase retardation image.

For 3D visualization, structural intensity images were compiled in Amira 3D visualizing software. In the rendered 3D images, the conduit structure was displayed in grayscale, while the nerve was visualized in color (**Figure 1A-C**). To enhance the visibility of the nerve within the conduit, the conduit intensity was rendered semi-transparent.

From the enface phase retardation image, the optimal nerve connection path was manually traced (**Figure S1**). Phase retardation along this path was measured by averaging the phase retardation values within a 150 µm diameter window centered on the traced path. For each rat, the mean phase retardation values were calculated over 1 mm long segments at the proximal and distal ends inside the conduit. The mean

and standard error of these measurements were used to quantitatively compare the three experimental groups (**Figure 1D**).

Histology

After PS-OCT imaging, the harvested tissues were sectioned using a cryostat (Leica) [S2]. Longitudinal nerve-conduit sections were immunohistochemically labeled with anti-SOX2 (Proteintech, 11064-1-AP), anti-p75NTR (Cell Signaling, 8238T), or anti-NRG1 (Proteintech, 10527-1-AP) antibodies, followed by secondary antibodies conjugated to horseradish peroxidase (Jackson ImmunoResearch) and DAB substrate (Fisher). Samples labeled with anti-p75NTR and anti-SOX2 were counterstained with hematoxylin to visualize cell nuclei and imaged using a bright-field microscope (Olympus). Quantification of nuclear SOX2 intensity, cytoplasmic P75-NTR, and NRG1 over the area of imaging fields was conducted using ImageJ software.

Cell culture

All experiments involving human stem cells were approved by the UC Riverside Institutional Review Board (IRB; HS11-124) and Stem Cell Research Oversight Committee (SCRO; SC20210002). Induced pluripotent stem cells (iPSCs) were derived from BJ-2522 human neonatal foreskin fibroblast cells (ATCC) and their pluripotency was confirmed [S6]. The normal karyotype of the iPSC line was confirmed. Cells were tested for mycoplasma contamination using a luminescence-based method with a Lonza Lucetta Luminometer authenticated by the Stem Cell Center in University of California, Riverside. There was no bacteria or fungus contamination observed under either the bright field microscope or the fluorescence microscope. Neural crest stem cells (NCSCs) were derived from iPSCs using a protocol described elsewhere [S7]. Briefly, passage-43 iPSCs were thawed in 37 °C bath and maintained in mTeSR1 (Stemcell technologies) media on Geltrex-coated 6-well plate (Falcon). Once confluent, cells were detached using Accutase (Fisher) and seeded onto electrospun P(VDF-TrFE) scaffolds at a density of 150,000 cells/scaffold (7 mm x 5 mm). The modified Dalton NCSC differentiation protocol was used to guide the differentiation of the seeded iPSCs into trunk NCSCs [S7, S8]. NCSC-like cells were then subjected to physical stimulation (MES), using electrospun P(VDF-TrFE) scaffolds activated by the hydroacoustic actuator as previously described [S1]. Biphasic electric pulses at 3 Hz with an amplitude of 200 mV_{p-p} were generated on the surface of P(VDF-TrFE) scaffolds under the hydroacoustic actuation, directly stimulating the cells. Biochemical stimulation (BC; NRG1, 10 ng/mL) or the combination of both (MES+BC) were also separately applied. Cells cultured on scaffolds without MES or BC were used as the Control group. The cells were stimulated for 2 hours for various overall culture durations including 0 week (cells were stimulated only once), 1 week, 2 weeks, 3 weeks, or 4 weeks before fixation in 4% PFA (Fisher) or lysed using RLT buffer (Qiagen) for imaging and gene expression analyses, respectively. For mechanistic studies, signaling inhibitors including Smad inhibitor (Dorsomorphin dihydrochloride, 5 µM, Tocris) and NRG1 inhibitor (Seribantumab, 5 µM, MedChemExpress) were used. These inhibitors were individually applied under the MES condition for a week before cell fixation or lysing.

Immunofluorescence imaging

Fixed cells were immune-stained with various markers including, anti-TUJ.1 (Fisher, MA1118), anti-GALC (Proteintech, 11991-1-AP), anti-NEUN (Abcam, ab177487), anti-NRG1, anti-smad1/5 (Santa Cruz Biotechnology, sc-7965, sc-101151), anti-p-Smad1/5/8 (Sigma, AB3848-I), and anti-NFATc3 (DSHB, PCRP-NFATC3-2A12), followed by appropriate secondary antibodies conjugated with fluorophores (Goat Anti-Mouse IgG (H+L) with Alexa Fluro@ 488 or 594 and Goat Anti-Rabbit IgG (H+L) with Alexa Fluro@ 488 or 594, Jackson ImmunoResearch). A confocal microscope (Zeiss) was used to image the expression of different markers. ImageJ software was used to quantify fluorescence intensity or protein nuclear localization. For the quantification of nuclear localization, the Coloc2 plugin in ImageJ was used and the resulting Pearson's coefficient value was used to describe the degree of NFATc3 nuclear localization. A value of +1 indicates complete nuclear localization, while -1 indicates complete cytoplasmic localization [S9-S11].

Gene expression analysis

Total RNA was extracted using an RNeasy Micro Kit (Qiagen) followed by cDNA synthesis using an iScript cDNA Synthesis Kit (Bio-rad). RT-qPCR was performed to determine the phenotypic expression of neuronal, astrocytic, and oligodendrocytic genes (**Table S1**). Raw data were analyzed by the comparative threshold cycle (C_T) method using the expression of Gapdh as an endogenous control.

Elisa assay

Elisa (Enzyme-linked immunosorbent assay) was used to assess extracellular NRG1 secretion. At 2 weeks under various cell culture conditions such as Control, BC, MES, and MES+BC conditions, supernatant was collected 0, 2, 4, 8, and 24 hours after the last daily stimulation. Elisa was performed following the manufacture protocol (R&D Systems).

Statistical analysis

All experiments were conducted with a minimum of triplicate biological samples and data are presented as mean ± standard error of means. Comparison of experimental groups for statistical significance was determined using the IBM SPSS software with either one-way ANOVA with Tukey's HSD post-hoc test or a two-sample student *T*-test. Statistical significance was reported when a '*p*' value was less than 0.05 and 0.01.

Supplemental references

S1. Tai, Y., Ico, G., Low, K., Liu, J., Jariwala, T., Garcia-Viramontes, D., Lee, K.H., Myung, N.V., Park, B.H., and Nam, J. (2021). Formation of 3D Self-Organized Neuron-Glial Interface Derived from Neural Stem Cells via Mechano-Electrical Stimulation. Adv Healthc Mater *10*, e2100806. 10.1002/adhm.202100806.

S2. Tai, Y., Tonmoy, T.I., Win, S., Brinkley, N.T., Park, B.H., and Nam, J. (2023). Enhanced peripheral nerve regeneration by mechano-electrical stimulation. NPJ Regen Med *8*, 57. 10.1038/s41536-023-00334-y.

S3. Mitsui, T. (1999). Dynamic range of optical reflectometry with spectral interferometry. Jpn J Appl Phys 1 38, 6133-6137. Doi 10.1143/Jjap.38.6133.

S4. Park, B.H., Saxer, C., Srinivas, S.M., Nelson, J.S., and de Boer, J.F. (2001). In vivo burn depth determination by high-speed fiber-based polarization sensitive optical coherence tomography. J Biomed Opt 6, 474-479. Doi 10.1117/1.1413208.

S5. Hee, M.R., Huang, D., Swanson, E.A., and Fujimoto, J.G. (1992). Polarization-Sensitive Low-Coherence Reflectometer for Birefringence Characterization and Ranging. J Opt Soc Am B 9, 903-908. Doi 10.1364/Josab.9.000903.

S6. Torrez, L.B., Perez, Y., Yang, J., Zur Nieden, N.I., Klassen, H., and Liew, C.G. (2012). Derivation of neural progenitors and retinal pigment epithelium from common marmoset and human pluripotent stem cells. Stem Cells Int 2012, 417865. 10.1155/2012/417865.

S7. Huang, M., Miller, M.L., McHenry, L.K., Zheng, T., Zhen, Q., Ilkhanizadeh, S., Conklin, B.R., Bronner, M.E., and Weiss, W.A. (2016). Generating trunk neural crest from human pluripotent stem cells. Sci Rep 6, 19727. 10.1038/srep19727.

S8. Menendez, L., Kulik, M.J., Page, A.T., Park, S.S., Lauderdale, J.D., Cunningham, M.L., and Dalton, S. (2013). Directed differentiation of human pluripotent cells to neural crest stem cells. Nat Protoc 8, 203-212. 10.1038/nprot.2012.156.

S9. Costes, S.V., Daelemans, D., Cho, E.H., Dobbin, Z., Pavlakis, G., and Lockett, S. (2004). Automatic and quantitative measurement of protein-protein colocalization in live cells. Biophys J 86, 3993-4003. 10.1529/biophysj.103.038422.

S10. Dunn, K.W., Kamocka, M.M., and McDonald, J.H. (2011). A practical guide to evaluating colocalization in biological microscopy. Am J Physiol Cell Physiol 300, C723-742. 10.1152/ajpcell.00462.2010.

S11. Manders, E.M.M., Verbeek, F.J., and Aten, J.A. (1993). Measurement of co-localization of objects in dual-colour confocal images. J Microsc 169, 375-382. 10.1111/j.1365-2818.1993.tb03313.x.
