# Supplementary material for: The regenerative role of neural crest stem cells in physical stimuli-enhanced peripheral nerve repair
Source: Stem Cell Reports. 2026 Mar 26;21(4):102861. doi: 10.1016/j.stemcr.2026.102861 (PMC13083801; doi:10.1016/j.stemcr.2026.102861)
Supplement: Document S2. Article plus supplemental information [file mmc2.pdf]

STEM CELL REPORTS

ISSCR

Report

OPEN ACCESS

The regenerative role of neural crest stem cells in physical stimuli-enhanced peripheral nerve repair

Youyi Tai,[1,2] Lu Jin,[1,2] Thamidul Islam Tonmoy,[1] B. Hyle Park,[1] and Jin Nam[1,3,*]
[1]Department of Bioengineering, University of California, Riverside, Riverside, CA 92521, USA
[2]These authors contributed equally
[3]Lead contact
*Correspondence: jinnam@ucr.edu
https://doi.org/10.1016/j.stemcr.2026.102861

SUMMARY

Neural crest stem cells (NCSCs), capable of differentiating into neurons and Schwann cells, are essential for peripheral nerve regeneration. This study investigates the role of endogenous NCSC-like cells in mechano-electrical stimulation (MES)-enhanced peripheral nerve repair. In a critical-sized nerve injury model, MES leads to complete nerve reconnection, accompanied by a significant increase in NCSC-like cells at the injury sites. *In vitro*, MES promotes the simultaneous differentiation of NCSC-like cells into neurons and Schwann cells, with elevated neuregulin 1 (NRG1) expression, a key factor in Schwann cell development. Mechanistically, MES activates BMP/Smad signaling, driving neuronal differentiation and subsequent NRG1 secretion, which in turn promotes Schwann cell maturation through the ErBB/NFAT pathway. These findings demonstrate that MES enhances peripheral nerve regeneration by activating and directing stem cell differentiation, supporting a novel therapeutic approach that utilizes physical stimulation for stem cell modulation for nerve repair.

INTRODUCTION

Neural crest stem cells (NCSCs) that involve in the early development of the nervous system are present in various tissues derived from the neural crest (Shakhova and Sommer, 2008). While not as actively engaged in tissue functions, these adult NCSC-like cells can be activated by injury, allowing them to differentiate into various cell types facilitating tissue repair (Kastriti et al., 2022). Moreover, following peripheral nerve injury, mature Schwann cells have shown to dedifferentiate to NCSC-like cells, playing various regenerative roles (Parfejevs et al., 2018a).

Despite the regenerative potential of NCSCs, peripheral nerve injuries, especially those involving critical-sized defects (referring to a nerve gap that cannot be naturally repaired), often face limited self-repair due to uncontrolled axonal sprouting (Modrak et al., 2020). Autologous nerve transplantation remains the gold standard for treating peripheral nerve injury, but it typically does not provide full functional recovery for critical-sized nerve repairs (Hussain et al., 2020). Nerve conduits have been explored as an alternative, providing structural support for nerve regeneration over short distances by minimizing uncontrolled axonal sprouting. However, their effectiveness diminishes with critical-sized defects due to their lack of bio-conduciveness (Manoukian et al., 2021).

Given the challenges of using biochemical factors in therapeutic applications due to their limitations in long-term effectiveness, physical stimulation has emerged as a promising alternative to promote peripheral nerve regeneration such as electrical stimulation and electro-mechanical stimulation (Song et al., 2016; Pi et al., 2023). While these physical stimuli have shown promising outcomes, the mechanisms underlying physical stimulation-induced nerve regeneration remain unclear. Therefore, in this study, we first highlighted that mechano-electrical stimulation (MES), generated by therapeutic shockwave-activated piezoelectric conduits, can effectively promote the regeneration of peripheral nerve injuries by recruiting NCSC-like cells. Our mechanistic investigation revealed that BMP-dependent p-Smad1/5/8 initiates the neuronal differentiation of NCSC-like cells, leading to neuregulin 1 (NRG1) secretion and subsequent Schwann cell development through ErBB signaling and NFATc3 transcriptional activation. Collectively, this study demonstrates that MES enhances the recruitment and differentiation/maturation of NCSC-like cells, leading to functional recovery in peripheral nerve injuries.

RESULTS

Using a rat peripheral nerve transection model with a 15 mm critical gap size, we have demonstrated the improved nerve reconnection and motor functional recovery under the MES by various behavior and physiological analysis (Tai et al., 2023). Electrospun poly(vinylidene fluoride-trifluoroethylene) (P(VDF-TrFE)) was employed as piezoelectric nerve conduits bridging the critical nerve gap; one group was subjected to the periodic application of therapeutic shockwave to elicit MES via the activation of the implanted conduit (MES) while the other group

Published by Elsevier Inc. on behalf of International Society for Stem Cell Research.
This is an open access article under the CC BY license (http://creativecommons.org/licenses/by/4.0/).

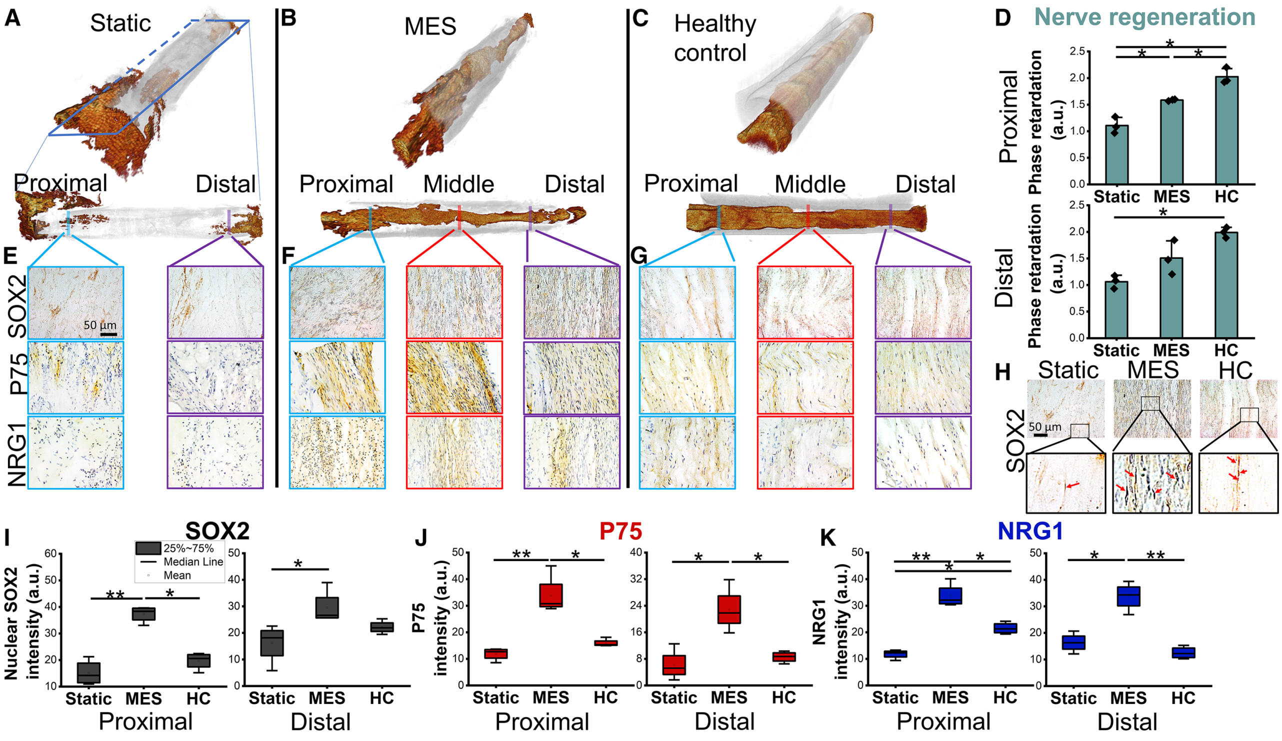

Figure 1. MES enhances peripheral nerve regeneration by recruiting NCSCs-like cells at the injury site

(A–C) Representative 3D reconstructed PS-OCT images of sciatic nerves (A) without or (B) with MES in a rat sciatic transection model. The nerve conduit is indicated by translucent gray while the nerve is indicated by red-brown color. A normal sciatic nerve wrapped in the conduit was used as a (C) HC. After conduit transplantation onto the transected sciatic nerve, the injury site was subjected to MES using therapeutic shockwave (1,000 pulses per time, twice a week) for 12 weeks. The PS-OCT images and immunohistochemistry staining were conducted 12 weeks post-surgery.

(D) Quantification of phase retardation of PS-OCT images at proximal and distal ends ($n = 3$, mean ± SEM).

(E–G) Representative immunohistochemical images showing the expression of neural crest markers SOX2, P75-NTR, and nerve regeneration factor NRG1 at the proximal/distal ends and the middle of the conduits bridging transected sciatic nerves under (E) static, (F) MES, or (G) HC conditions.

(H) Representative zoomed in images of nuclear SOX2.

(I–K) Quantification of histology staining intensities of (I) nuclear SOX2, (J) P75, and (K) NRG1. * and ** denote statistical significance of $p < 0.05$ and $p < 0.01$. After conduit transplantation onto the transected sciatic nerve, the injury site was subjected to MES using therapeutic shockwave (1,000 pulses per time, twice a week) for 12 weeks. The PS-OCT images and immunohistochemistry staining were conducted 12 weeks post-surgery. Quantification data were collected from a total of 9 images from 3 rats for each condition.

was not subjected to exogenous stimulation (Static). After 12 weeks post-implantation, polarization-sensitive optical coherence tomography (PS-OCT) was employed to non-destructively assess nerve regeneration (Figures 1A–1C). The implantation of the piezoelectric P(VDF-TrFE) conduit did not result in the reconnection of the transected nerves with a critical nerve gap (Figure 1A). In contrast, 12 weeks of MES treatment led to full nerve reconnection (Figure 1B), closely resembling the structure of a healthy sciatic nerve (Figure 1C). The phase retardation value, a quantification of optical birefringence passing through nerve tissue indicating nerve structure integrity (Tai et al., 2023), under the MES condition was significantly greater at the proximal end as compared to the Static condition and approached that of the healthy control (HC) at the distal end (Figures 1D and S1). In this study, under the MES condition, we observed an increased cell population expressing SOX2, a marker for NCSCs, indicating an active regeneration process (Johnston et al., 2016), as compared to the Static and Healthy controls (Figures 1E–1I). Another NCSC marker, p75 neurotrophic receptor (p75NTR), was substantially expressed under the MES condition, likely indicating the increased population of NCSC-like cells promoted by MES at the injury sites (Figures 1E–1G and 1J). Corroborating with the increased expression of SOX2 and p75NTR, the expression of NRG1, a potent neurotrophic factor during peripheral nerve regeneration (Tseropoulos et al., 2024), was also enhanced under MES (Figure 1K).

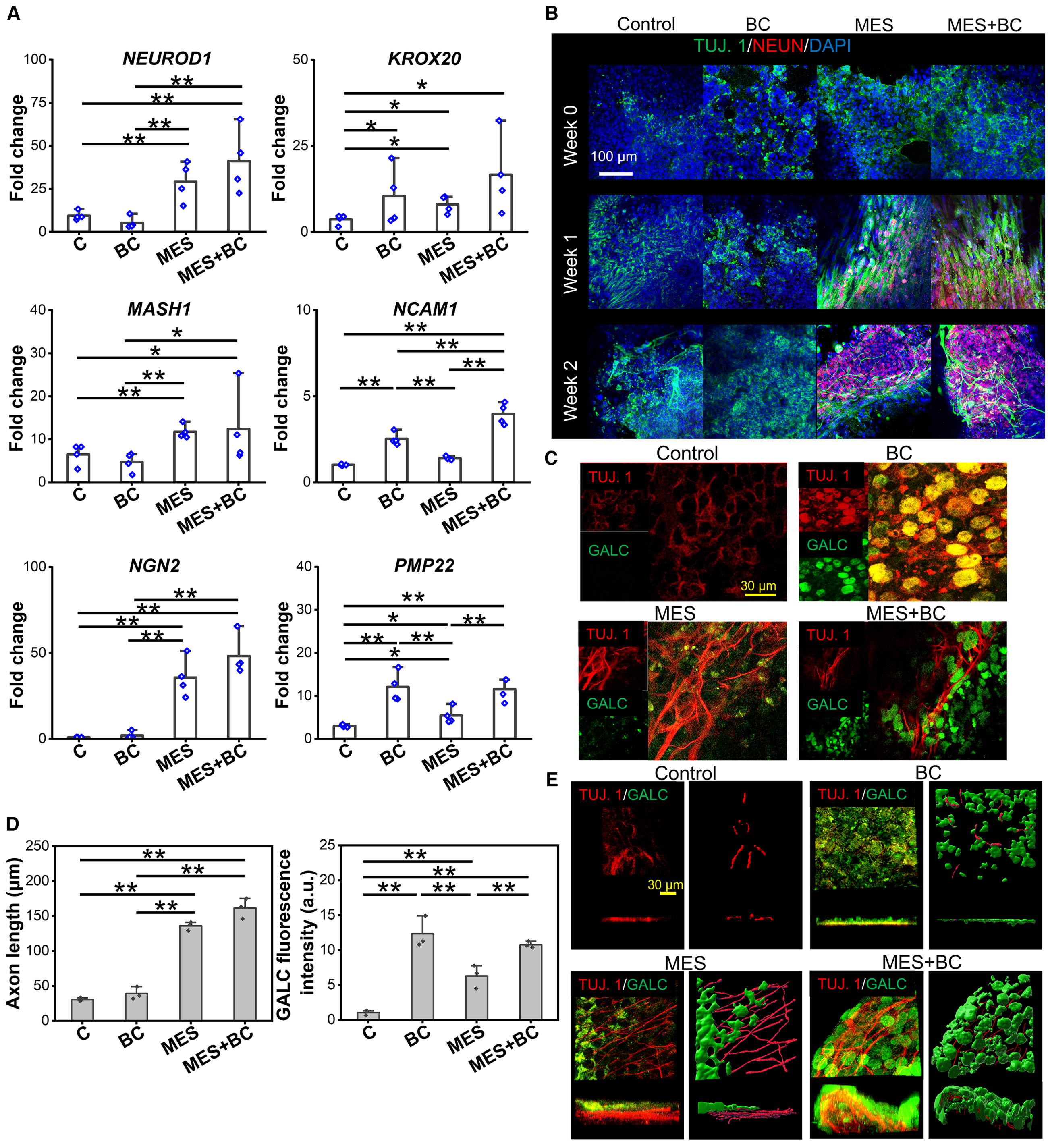

Figure 2. MES induces the multiphenotypic differentiation of NCSCs-like cells toward neurons and Schwann cells *in vitro*

(A) Gene expression of neuronal markers *NEUROD1*, *MASH1*, *NGN2*, and Schwann cell markers *KROX20*, *NCAM1*, *PMP22* after 1 week of culture under the control (C), biochemical factor (BC), MES, and MES+BC conditions. $n = 4$ (biologically independent), mean ± SEM.

(B) Confocal images showing the expression of neuronal markers (beta III tubulin [TUJ. 1] and NEUN) after 0, 1, and 2 weeks of culture under the control, BC, MES, and MES+BC conditions.

(C) Confocal images showing the expression of a neuronal marker TUJ. 1, a Schwann cell marker GALC after 2 weeks of culture under the control, BC, MES, and MES+BC conditions.

(D) Quantification of axon length and GALC fluorescence intensity. $n = 3$ (biologically independent), mean ± SEM.

(legend continued on next page)

To investigate the potential mechanisms of NCSC-like cells-mediated nerve regeneration under MES, the cellular responses of NCSCs, derived from human-induced pluripotent stem cells (iPSCs) (Figure S2), were investigated under MES *in vitro*. The role of NRG1, which was upregulated during nerve regeneration in our animal study as well as reported by others (Nocera and Jacob, 2020), was also examined on the neuro-regenerative behaviors of NCSC-like cells by supplementing it as an additional biochemical factor (BC) in combination with MES. The MES condition upregulated neurogenetic genes, including *NEUROD1*, *MASH1*, and *NGN2*, while the BC condition upregulated Schwann cell development genes, such as *KROX20*, *NCAM1*, and *PMP22* (Figure 2A). Interestingly, the combination of MES and BC significantly induced the upregulation of both genes related to neurogenesis and Schwann cell development. The culture duration-dependent protein expression of neuronal markers was examined under various conditions (Figures 2B and S3); results showed that βIII-tubulin (TUJ. 1) expression was presented in all conditions, consistent with the previous research reporting that trunk NCSCs express βIII-tubulin (Chacon and Rogers, 2019). However, a distinct morphological change of TUJ. 1^+ cells was observed under MES (MES and MES+BC conditions), showing somas with elongated neurites at week 1 and more extended neuronal projections at week 2, in comparison to a round stem cell-like phenotype under the control or BC alone conditions. The neuronal differentiation of NCSC-like cells under MES was further confirmed by the presence of mature neuronal marker NEUN. Different from the expression of neuronal markers, a stronger GALC expression, a marker for Schwann cell differentiation, was observed under NRG1 supplementation (BC and MES+BC conditions) (Figure 2C), consistent with the gene expression results (Figure 2A). Overall, there was a significantly enhanced axonal extension under the MES-involved conditions, while a stronger GALC expression was observed under the BC-involved conditions (Figure 2D). Furthermore, an extended culture duration (4 weeks) induced a 3D tissue-like structure in both MES and MES+BC conditions; the MES condition exhibited a layered cell structure with Schwann cells populated on top of neurons, whereas the MES+BC condition resulted in the tubular nerve bundle formation with individual Schwann cell wrapping around the axons (Figure 2E). In contrast, the control and BC conditions led to a single-layered, 2D tissue formation.

Previous studies have shown that BMP4/Smad and NRG1/ErBB/NFATc3 signaling cascades are involved in the neuronal differentiation and Schwann cell development of NCSCs, respectively (Kao et al., 2009; Mehler et al., 1997). Therefore, we examined whether MES induces the multiphenotypic differentiation of NCSC-like cells through the activation of these signaling pathways. While Smad1/5 expression was observed in all conditions (Figure S4), nuclear localization of p-Smad1/5/8 was observed only under MES and MES+BC conditions throughout the culture period (Figures 3A and 3B), indicating the activation of the BMP signaling by MES. The application of dorsomorphin, a Smad inhibitor, resulted in the suppression of the MES-upregulated expression of early neuronal markers (*NEUROD1*, *MASH1*, and *NGN2*) while the expression of Schwann cell developmental genes (*KROX20*, *NCAM1*, and *PMP22*) was not influenced by the inhibition of the Smad signaling cascade (Figure 3C).

In regard to the expression pattern of NFATc3, a crucial signaling pathway governing Schwann cell development (Kao et al., 2009), both BC and MES+BC exhibited a significant increase in nuclear localization, as compared to the control and MES conditions at week 0 and week 1 (Figures 4A and 4B). A lack of nuclear localization of NFATc3, observed under the MES condition at week 0 and week 1, drastically changed at week 2, showing a significant nuclear translocation. Since NRG1 has been shown to activate the NFATc3 signaling, we then determined whether the expression of endogenous NRG1 was regulated by physical stimulation. Because NRG1 is primarily expressed by mature neurons, the week 2 time point, when NEUN expression emerged under MES (Figure 2B), was used in the subsequent studies. As expected, the gene expression of *NRG1* was upregulated under the BC, MES, and MES+BC conditions (Figure 4C). The MES+BC condition, however, induced a significantly greater upregulation compared with other conditions, potentially indicating a synergistic effect of BC and MES in regulating NRG1. Similarly, significantly increased NRG1 expression was observed in immunofluorescence imaging under the MES+BC condition (Figures 4D and 4E), demonstrating the inductive role of physical stimulation on NRG1 secretion. The increased endogenous NRG1 expression under the BC condition could be attributed to the positive feedback loop of NRG1 regulation (Frensing et al., 2008). The extracellular secretion of NRG1 similarly showed significantly increased levels under the BC, MES, and

(E) Confocal images and corresponding Imaris 3D reconstruction images of the cells after 4 weeks of culture under the control, BC, MES, and MES+BC conditions, the cells were fluorescently labeled by TUJ. 1 and GALC. NCSC-like cells were subjected to acoustic actuator stimulation as MES, biochemical factor NRG1 stimulation as BC, or the combination of both as MES+BC. The cells were stimulated for 2 h daily for either 2 weeks or 4 weeks * and ** denote statistical significance of $p < 0.05$ and $p < 0.01$, respectively. * and ** denote statistical significance $p < 0.05$ and $p < 0.01$, respectively.

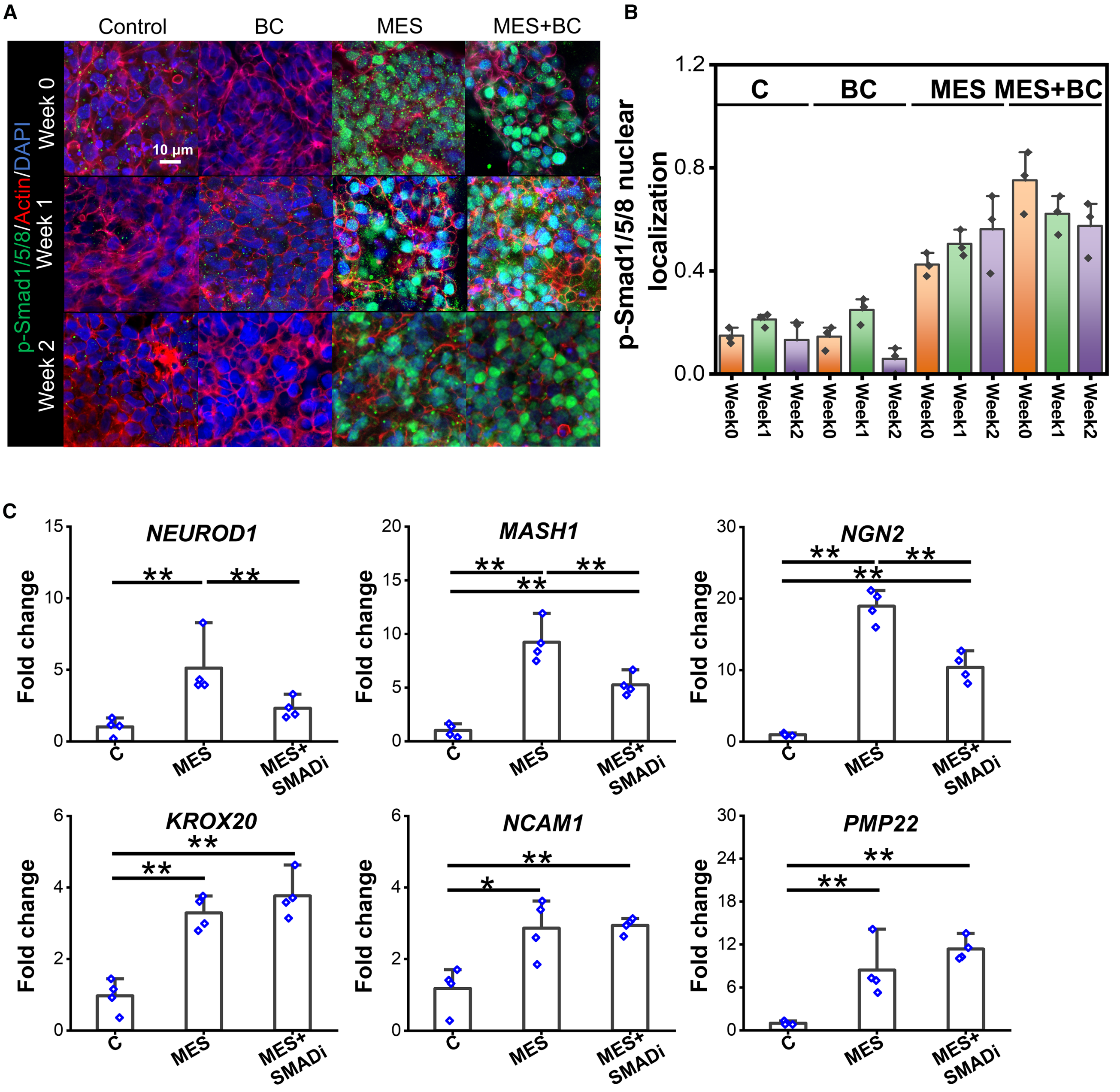

Figure 3. The role of p-Smad in MES-mediated NCSCs-like cells differentiation

(A) Confocal images showing the expression of p-Smad1/5/8 after 0, 1 week, and 2 weeks of culture under the control, biochemical factor (BC), MES, and MES+BC conditions. The cells were stimulated for 2 h daily for either 0 week (2-h stimulation was applied to the cells only once), 1 week, or 2 weeks before sample fixation.

(B) Quantification of nuclear localization of p-Smad1/5/8. $n = 3$ (biologically independent), mean ± SEM.

(C) Gene expression levels of neuronal markers *NEUROD1*, *MASH1*, *NGN2*, and Schwann cell markers *KROX20*, *NCAM1*, and *PMP22* after the 1-week application of MES in the presence of BMP-dependent Smad inhibitor, dorsomorphin dihydrochloride (SMADi). The control (S) and MES without any inhibitors were included as negative and positive controls, respectively. $n = 4$ (biologically independent), mean ± SEM. * and ** denote statistical significance of $p < 0.05$ and $p < 0.01$, respectively.

MES+BC conditions (Figure 4F). Interestingly, MES and MES+BC exhibited an increasing NRG1 secretion trend while it remained at a similar level throughout the entire duration under the BC condition. Moreover, the inhibition of NRG1 by seribantumab significantly downregulated Schwann cell differentiation while neuronal

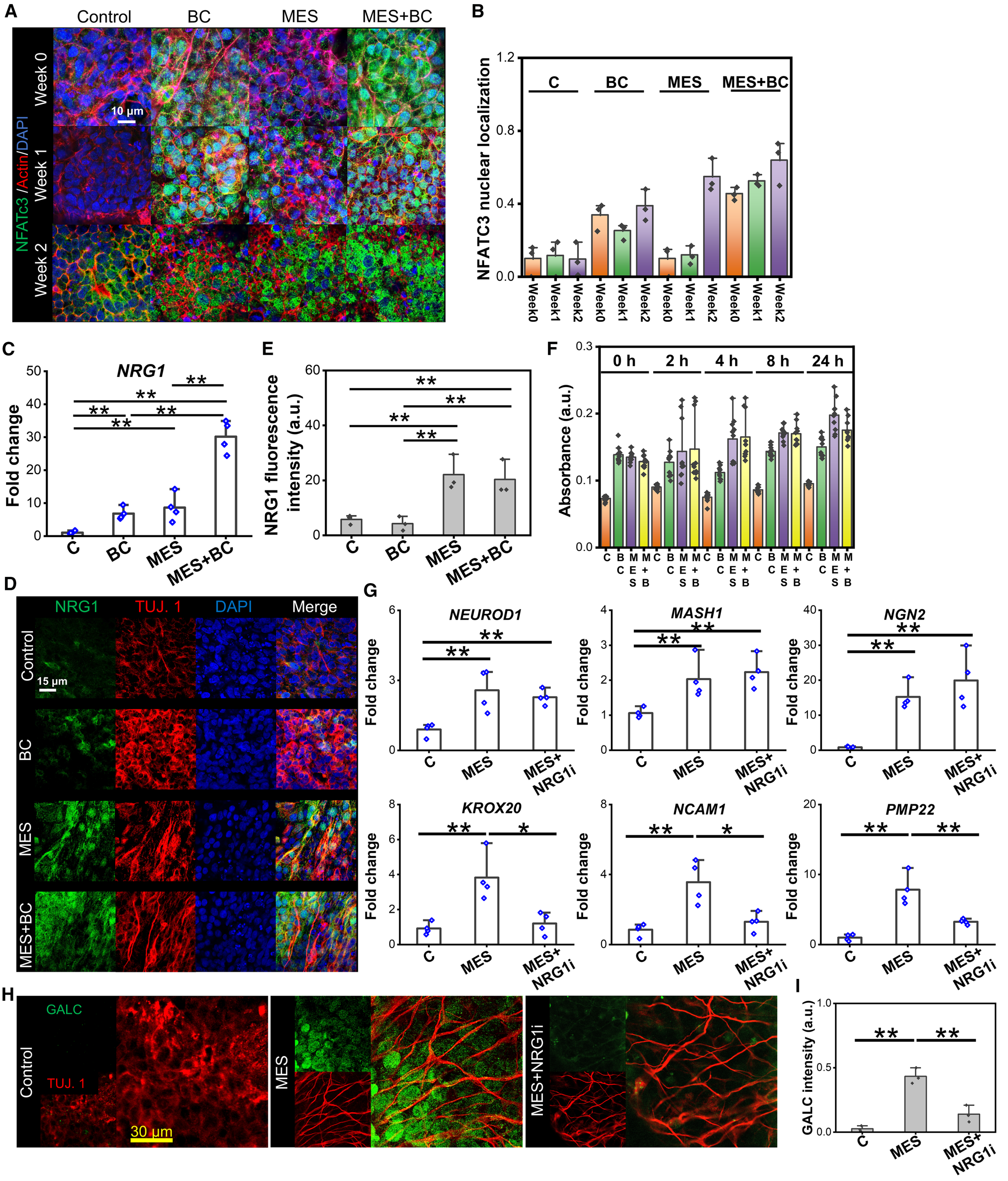

Figure 4. The role of NRG1/NFATc3 cascade in MES-mediated NCSCs-like cells differentiation

(A) Confocal images showing the expression of NFATc3 after 0, 1 week, and 2 weeks of culture under the control (C), biochemical factor (BC), MES, and MES+BC conditions.

(B) Quantification of nuclear localization of NFATc3. $n = 3$ (biologically independent), mean ± SEM.

(legend continued on next page)

differentiation was not affected (Figure 4G). These gene expression results were consistent at the protein level, where MSE under the treatment with NRG1 inhibitor decreased the expression of GALC, a mature Schwann cell marker (Figures 4H and 4I).

DISCUSSION

During peripheral nerve regeneration, Schwann cells de-differentiate to non-myelinating Schwann precursors that share similar cellular characteristics with NCSCs (Parfejevs et al., 2018b). These NCSC-like cells have been shown to function as repair cells to facilitate peripheral nerve regeneration by supporting the regrowth of damaged axons and reforming myelinating Schwann cells (Balakrishnan et al., 2021; Parfejevs et al., 2018a). Interestingly, our animal study, where piezoelectric conduit-mediated physical stimulation enhanced peripheral nerve regeneration, showed emergence of such a cell population with SOX2 and p75NTR expression (Johnston et al., 2013, 2016; Lee et al., 2007), suggesting the effect of MES in recruiting/generating NCSC-like cells at the injury site. Furthermore, the increased level of neurotrophic NRG1 expression in these cells indicates the involvement of NCSC-like cells in nerve regeneration in response to physical stimulation (Birchmeier and Nave, 2008; El Soury and Gambarotta, 2019; Tseropoulos et al., 2024).

To understand the implication of the emergence of NCSC-like cells under MES *in vivo*, the effects of MES on NCSC-like cells were further examined *in vitro*. MES induced the multiphenotypic differentiation of NCSC-like cells toward neurons and Schwann cells. Interestingly, the supplementation of NRG1, which was upregulated *in vivo* under MES, further augmented the formation of a unique 3D tissue structure. This structure is similar to the defining characteristics of the peripheral nerve where neurons are myelinated by individual Schwann cells along axons (Melemedjian and Khoutorsky, 2015). These results may suggest that endogenous NCSC-like cells actively participate in peripheral nerve regeneration by differentiating to appropriate cell phenotypes under physical stimulation.

Consistent with the increased expression of NRG1 observed in regenerating nerves *in vivo*, MES alone was sufficient to upregulate NRG1 expression *in vitro*. MES induced extended axon formation, leading to the upregulation of NRG1, which is richly expressed in mature axons (Brinkmann et al., 2008). BMP4/Smad signaling has been shown to regulate neuronal differentiation in the peripheral nerve system (Mehler et al., 1997) while our study demonstrated the Smad activation by MES. Although our present study did not reveal the direct linkage between MES and the BMP signaling, several previous studies have found that physical cues regulate the BMP signaling cascade through various mechanisms, including increased BMP protein expression (Sato et al., 1999), enhanced focal adhesion formation that leads to BMP receptor activation (Görlitz et al., 2024), and increased efficiency of Smad phosphorylation (Kido et al., 2010). Similarly, electrical stimulation has also been shown to directly increase the BMP protein level, initiating the signaling cascade (Srirussamee et al., 2019). Nevertheless, our results suggest that MES enhances neuronal differentiation/maturation of NCSC-like cells, resulting in the upregulation of NRG1. NRG1 binds to ErBB channels, activating calcineurin/NFAT signaling that drives Schwann cell differentiation/maturation (Kao et al., 2009). Our study proved that NRG1 inhibition leads to a decreased nuclear expression of NFATc3 (Figure S5), indicating the role of NRG1 in bridging between neuronal maturation and Schwann cell development.

In summary, our study demonstrates the recruitment of endogenous NCSC-like cells for peripheral nerve regeneration, in response to physical stimulation. Our mechanistic study revealed that MES induces neuronal differentiation

(C) Gene expression level of *NRG1* after 2 weeks of culture under the S, BC, MES, and MES+BC conditions. $n = 4$ (biologically independent).
(D) Confocal images showing the intracellular NRG1 and beta III tubulin expression after 2 weeks of culture under various conditions.
(E) Quantification of NRG1 fluorescence intensity and ratio of $NRG1^+$ cell population based on the images in (D). $n = 3$ (biologically independent).
(F) Elisa assay results indicating the extracellular NRG1 secretion levels after 2 weeks of culture under the S, BC, MES, and MES+BC conditions. For this experiment, cell supernatants under various conditions after 2 weeks of culture was collected 0, 2, 4, 8, and 24 h after the last stimulation. $n = 10$ (5 biologically independent samples, 2 technical duplicates).
(G) Gene expression levels of neuronal markers *NEUROD1*, *MASH1*, *NGN2*, and Schwann cell markers *KROX20*, *NCAM1*, and *PMP22* after the 3-week application of MES in the presence of NRG1 inhibitor, seribantumab (NRG1i). The control and MES without any inhibitors were included as negative and positive controls, respectively. $n = 4$ (biologically independent), mean ± SEM.
(H) Confocal images showing the expression of a neuronal marker TUJ. 1, a Schwann cell marker GALC after 3 weeks of culture under the control, MES, and NRG1i conditions.
(I) Quantification of GALC fluorescence intensity. $n = 3$ (biologically independent), mean ± SEM. * and ** denote statistical significance of $p < 0.05$ and $p < 0.01$, respectively. NCSC-like cells were subjected to acoustic actuator stimulation as MES, biochemical factor NRG1 stimulation as BC, or the combination of both as MES+BC under various culture durations.

and maturation of NCSC-like cells through the activation of BMP/Smad signaling, leading to the secretion of neurotrophic factor NRG1. This, in turn, activates the ErBB/NFATc3 signaling cascade, resulting in Schwann cell differentiation (Figure S6). This study provides a strong basis for developing therapies for nerve injuries through the combination of patient-derived NCSC-like cells and physical stimulation for enhanced regenerative outcomes.

METHODS

All procedures involving animals and human cells were approved by appropriate institutional committee (IACUC: 20210016; IRB: HS11-124; SCRO: SC20210002). Detailed experimental methods are presented in supplemental information.

Electrospinning of P(VDF-TrFE) scaffolds

P(VDF-TrFE) fibers were fabricated as previously described (Tai et al., 2021). Electrospun fibers having an average diameter of 500 nm were collected on a rotating wheel (47.9 m/s) to form aligned scaffolds (~200 μm thick), followed by annealing at 90°C for 24 h.

Animal surgery

Sciatic nerve transection (15 mm gap) was performed in adult Sprague-Dawley rats ($n = 9$), with P(VDF-TrFE) conduits sutured to nerve stumps. MES group ($n = 3$) received shockwave stimulation twice weekly; Static group ($n = 3$) received none. At 12 weeks, rats were euthanized, and conduits with bridging nerves were harvested for subsequent imaging analyses.

PS-OCT imaging

Fixed tissues were imaged using a custom spectral domain PS-OCT system (Tai et al., 2023). Structural and phase retardation volumes were reconstructed using MATLAB and visualized in Amira.

Histology

Cryosectioned nerve conduits were immunolabeled with anti-SOX2, p75NTR, or NRG1, followed by HRP-conjugated secondaries and DAB staining. Nuclei were counterstained with hematoxylin and imaged by bright-field microscopy.

Cell culture and stimulation

NCSCs derived from iPSCs (Huang et al., 2016) were cultured on P(VDF-TrFE) scaffolds under control, MES, BC (NRG1, 10 ng/mL), or MES+BC conditions. Stimulation (2 h/day) was applied for up to 4 weeks. For mechanistic studies, cells were treated with Smad (Dorsomorphin, 5 μM) or NRG1 (Seribantumab, 5 μM) inhibitors under MES.

Immunofluorescence

Fixed cells were stained with TUJ1, GALC, NEUN, NRG1, p-Smad1/5/8, and NFATc3, followed by secondary antibodies. Confocal microscopy was used for imaging with ImageJ for subsequent quantification.

RT-qPCR

Total RNA was extracted and reversely transcribed. Gene expression was quantified by RT-qPCR using GAPDH for normalization (Table S1).

ELISA

NRG1 secretion was quantified in supernatants collected at various timepoints (0–24 h post-stimulation) using an ELISA kit.

Statistical analysis

Data are presented as mean ± SEM. Statistical significance was assessed using one-way ANOVA with Tukey's post hoc or Student's t test (SPSS); $p < 0.05$ was considered significant.

RESOURCE AVAILABILITY

Lead contact

Requests for further information and resources should be directed to and will be fulfilled by the lead contact, Jin Nam (jinnam@ucr.edu).

Materials availability

This study did not generate new unique reagents.

Data and code availability

All data reported in this paper will be shared by the lead contact upon request.

ACKNOWLEDGMENTS

This study was partially supported by the National Science Foundation (CBET-1805975). Y.T. and L.J. were supported by a TRANSCEND fellowship from the California Institute for Regenerative Medicine (EDUC4-12752). The contents of this publication are solely the responsibility of the authors and do not necessarily represent the official view of CIRM or other agencies of the State of California.

AUTHOR CONTRIBUTIONS

Y.T., writing – review and editing, writing – original draft, methodology, investigation, formal analysis, and data curation; L.J., writing – visualization, methodology, investigation, formal analysis, and data curation; T.I.T., writing – original draft, investigation, formal analysis, and data curation; B.H.P., writing – review and editing, methodology, supervision, resources, funding acquisition, formal analysis, and data curation; J.N., writing – review and editing, writing – original draft, validation, supervision, resources,

project administration, funding acquisition, formal analysis, data curation, and conceptualization.

DECLARATION OF INTERESTS

The authors declare no conflict of interest.

SUPPLEMENTAL INFORMATION

Supplemental information can be found online at https://doi.org/10.1016/j.stemcr.2026.102861.

Received: July 1, 2025
Revised: February 23, 2026
Accepted: February 24, 2026
Published: March 26, 2026

REFERENCES

Balakrishnan, A., Belfiore, L., Chu, T.H., Fleming, T., Midha, R., Biernaskie, J., and Schuurmans, C. (2020). Insights Into the Role and Potential of Schwann Cells for Peripheral Nerve Repair From Studies of Development and Injury. Front. Mol. Neurosci. *13*, 608442. https://doi.org/10.3389/fnmol.2020.608442.

Birchmeier, C., and Nave, K.A. (2008). Neuregulin-1, a Key Axonal Signal that Drives Schwann Cell Growth and Differentiation. Glia *56*, 1491–1497. https://doi.org/10.1002/glia.20753.

Brinkmann, B.G., Agarwal, A., Sereda, M.W., Garratt, A.N., Müller, T., Wende, H., Stassart, R.M., Nawaz, S., Humml, C., Velanac, V., et al. (2008). Neuregulin-1/ErbB signaling serves distinct functions in myelination of the peripheral and central nervous system. Neuron *59*, 581–595. https://doi.org/10.1016/j.neuron.2008.06.028.

Chacon, J., and Rogers, C.D. (2019). Early expression of Tubulin Beta-III in avian cranial neural crest cells. Gene Expr. Patterns *34*, 119067. https://doi.org/10.1016/j.gep.2019.119067.

El Soury, M., and Gambarotta, G. (2019). Soluble neuregulin-1 (NRG1): a factor promoting peripheral nerve regeneration by affecting Schwann cell activity immediately after injury. Neural Regen. Res. *14*, 1374–1375. https://doi.org/10.4103/1673-5374.253516.

Frensing, T., Kaltschmidt, C., and Schmitt-John, T. (2008). Characterization of a neuregulin-1 gene promoter:: Positive regulation of type I isoforms by NF-κB. Bba-Gene Regul. Mech. *1779*, 139–144. https://doi.org/10.1016/j.bbagrm.2007.11.007.

Görlitz, S., Brauer, E., Günther, R., Duda, G.N., Knaus, P., and Petersen, A. (2024). Temporal regulation of BMP2 growth factor signaling in response to mechanical loading is linked to cytoskeletal and focal adhesion remodeling. Commun. Biol. *7*, 1064. https://doi.org/10.1038/s42003-024-06753-x.

Huang, M., Miller, M.L., McHenry, L.K., Zheng, T., Zhen, Q., Ilkhanizadeh, S., Conklin, B.R., Bronner, M.E., and Weiss, W.A. (2016). Generating trunk neural crest from human pluripotent stem cells. Sci. Rep. *6*, 19727. https://doi.org/10.1038/srep19727.

Hussain, G., Wang, J., Rasul, A., Anwar, H., Qasim, M., Zafar, S., Aziz, N., Razzaq, A., Hussain, R., de Aguilar, J.L.G., and Sun, T. (2020). Current Status of Therapeutic Approaches against Peripheral Nerve Injuries: A Detailed Story from Injury to Recovery. Int. J. Biol. Sci. *16*, 116–134. https://doi.org/10.7150/ijbs.35653.

Johnston, A.P.W., Naska, S., Jones, K., Jinno, H., Kaplan, D.R., and Miller, F.D. (2013). Sox2-mediated regulation of adult neural crest precursors and skin repair. Stem Cell Rep. *1*, 38–45. https://doi.org/10.1016/j.stemcr.2013.04.004.

Johnston, A.P.W., Yuzwa, S.A., Carr, M.J., Mahmud, N., Storer, M.A., Krause, M.P., Jones, K., Paul, S., Kaplan, D.R., and Miller, F.D. (2016). Dedifferentiated Schwann Cell Precursors Secreting Paracrine Factors Are Required for Regeneration of the Mammalian Digit Tip. Cell Stem Cell *19*, 433–448. https://doi.org/10.1016/j.stem.2016.06.002.

Kao, S.C., Wu, H., Xie, J., Chang, C.P., Ranish, J.A., Graef, I.A., and Crabtree, G.R. (2009). Calcineurin/NFAT signaling is required for neuregulin-regulated Schwann cell differentiation. Science *323*, 651–654. https://doi.org/10.1126/science.1166562.

Kastriti, M.E., Faure, L., Von Ahsen, D., Bouderlique, T.G., Boström, J., Solovieva, T., Jackson, C., Bronner, M., Meijer, D., Hadjab, S., et al. (2022). Schwann cell precursors represent a neural crest-like state with biased multipotency. EMBO J. *41*, e108780. https://doi.org/10.15252/embj.2021108780.

Kido, S., Kuriwaka-Kido, R., Umino-Miyatani, Y., Endo, I., Inoue, D., Taniguchi, H., Inoue, Y., Imamura, T., and Matsumoto, T. (2010). Mechanical stress activates Smad pathway through PKCδ to enhance interleukin-11 gene transcription in osteoblasts. PLoS One *5*, e13090.

Lee, G., Kim, H., Elkabetz, Y., Al Shamy, G., Panagiotakos, G., Barberi, T., Tabar, V., and Studer, L. (2007). Isolation and directed differentiation of neural crest stem cells derived from human embryonic stem cells. Nat. Biotechnol. *25*, 1468–1475. https://doi.org/10.1038/nbt1365.

Manoukian, O.S., Rudraiah, S., Arul, M.R., Bartley, J.M., Baker, J.T., Yu, X., and Kumbar, S.G. (2021). Biopolymer-nanotube nerve guidance conduit drug delivery for peripheral nerve regeneration: In vivo structural and functional assessment. Bioact. Mater. *6*, 2881–2893. https://doi.org/10.1016/j.bioactmat.2021.02.016.

Mehler, M.F., Mabie, P.C., Zhang, D., and Kessler, J.A. (1997). Bone morphogenetic proteins in the nervous system. Trends Neurosci. *20*, 309–317. https://doi.org/10.1016/s0166-2236(96)01046-6.

Melemedjian, O.K., and Khoutorsky, A. (2015). Translational control of chronic pain. Prog. Mol. Biol. Transl. Sci. *131*, 185–213. https://doi.org/10.1016/bs.pmbts.2014.11.006.

Modrak, M., Talukder, M.A.H., Gurgenashvili, K., Noble, M., and Elfar, J.C. (2020). Peripheral nerve injury and myelination: Potential therapeutic strategies. J. Neurosci. Res. *98*, 780–795. https://doi.org/10.1002/jnr.24538.

Nocera, G., and Jacob, C. (2020). Mechanisms of Schwann cell plasticity involved in peripheral nerve repair after injury. Cell. Mol. Life Sci. *77*, 3977–3989.

Parfejevs, V., Antunes, A.T., and Sommer, L. (2018). Injury and stress responses of adult neural crest-derived cells. Dev. Biol. *444*, S356–S365. https://doi.org/10.1016/j.ydbio.2018.05.011.

Parfejevs, V., Debbache, J., Shakhova, O., Schaefer, S.M., Glausch, M., Wegner, M., Suter, U., Riekstina, U., Werner, S., and Sommer, L. (2018). Injury-activated glial cells promote wound healing of

the adult skin in mice. Nat. Commun. *9*, 236. https://doi.org/10.1038/s41467-017-01488-2.

Pi, W., Rao, F., Cao, J., Zhang, M., Chang, T., Han, Y., Zheng, Y., Liu, S., Li, Q., Sun, X., and Shao, Y. (2023). Sono-electro-mechanical therapy for peripheral nerve regeneration through piezoelectric nanotracts. Nano Today *50*, 101860. https://doi.org/10.1016/j.nantod.2023.101860.

Sato, M., Ochi, T., Nakase, T., Hirota, S., Kitamura, Y., Nomura, S., and Yasui, N. (1999). Mechanical tension-stress induces expression of bone morphogenetic protein (BMP)-2 and BMP-4, but not BMP-6, BMP-7, and GDF-5 mRNA, during distraction osteogenesis. J. Bone Miner. Res. *14*, 1084–1095. https://doi.org/10.1359/jbmr.1999.14.7.1084.

Shakhova, O., and Sommer, L. (2008). Neural crest-derived stem cells. In StemBook, F. Gage and F. Watt, eds. (Harvard Stem Cell Institude). https://doi.org/10.3824/stembook.1.51.1.

Song, J., Sun, B., Liu, S., Chen, W., Zhang, Y., Wang, C., Mo, X., Che, J., Ouyang, Y., Yuan, W., and Fan, C. (2016). Polymerizing Pyrrole Coated Poly (l-lactic acid-co-epsilon-caprolactone) (PLCL) Conductive Nanofibrous Conduit Combined with Electric Stimulation for Long-Range Peripheral Nerve Regeneration. Front. Mol. Neurosci. *9*, 117. https://doi.org/10.3389/fnmol.2016.00117.

Srirussamee, K., Mobini, S., Cassidy, N.J., and Cartmell, S.H. (2019). Direct electrical stimulation enhances osteogenesis by inducing Bmp2 and Spp1 expressions from macrophages and preosteoblasts. Biotechnol. Bioeng. *116*, 3421–3432. https://doi.org/10.1002/bit.27142.

Tai, Y., Ico, G., Low, K., Liu, J., Jariwala, T., Garcia-Viramontes, D., Lee, K.H., Myung, N.V., Park, B.H., and Nam, J. (2021). Formation of 3D Self-Organized Neuron-Glial Interface Derived from Neural Stem Cells via Mechano-Electrical Stimulation. Adv. Healthc. Mater. *10*, e2100806. https://doi.org/10.1002/adhm.202100806.

Tai, Y., Tonmoy, T.I., Win, S., Brinkley, N.T., Park, B.H., and Nam, J. (2023). Enhanced peripheral nerve regeneration by mechano-electrical stimulation. NPJ Regen. Med. *8*, 57. https://doi.org/10.1038/s41536-023-00334-y.

Tseropoulos, G., Mehrotra, P., Podder, A.K., Wilson, E., Zhang, Y., Wang, J., Koontz, A., Gao, N.P., Gunawan, R., Liu, S., et al. (2024). Immobilized NRG1 Accelerates Neural Crest like Cell Differentiation Toward Functional Schwann Cells Through Sustained Erk1/2 Activation and YAP/TAZ Nuclear Translocation. Adv. Sci. *11*, e2402607. https://doi.org/10.1002/advs.202402607.

Stem Cell Reports, Volume *21*

Supplemental Information

The regenerative role of neural crest stem cells in physical stimuli-enhanced peripheral nerve repair

Youyi Tai, Lu Jin, Thamidul Islam Tonmoy, B. Hyle Park, and Jin Nam

Supplemental figures

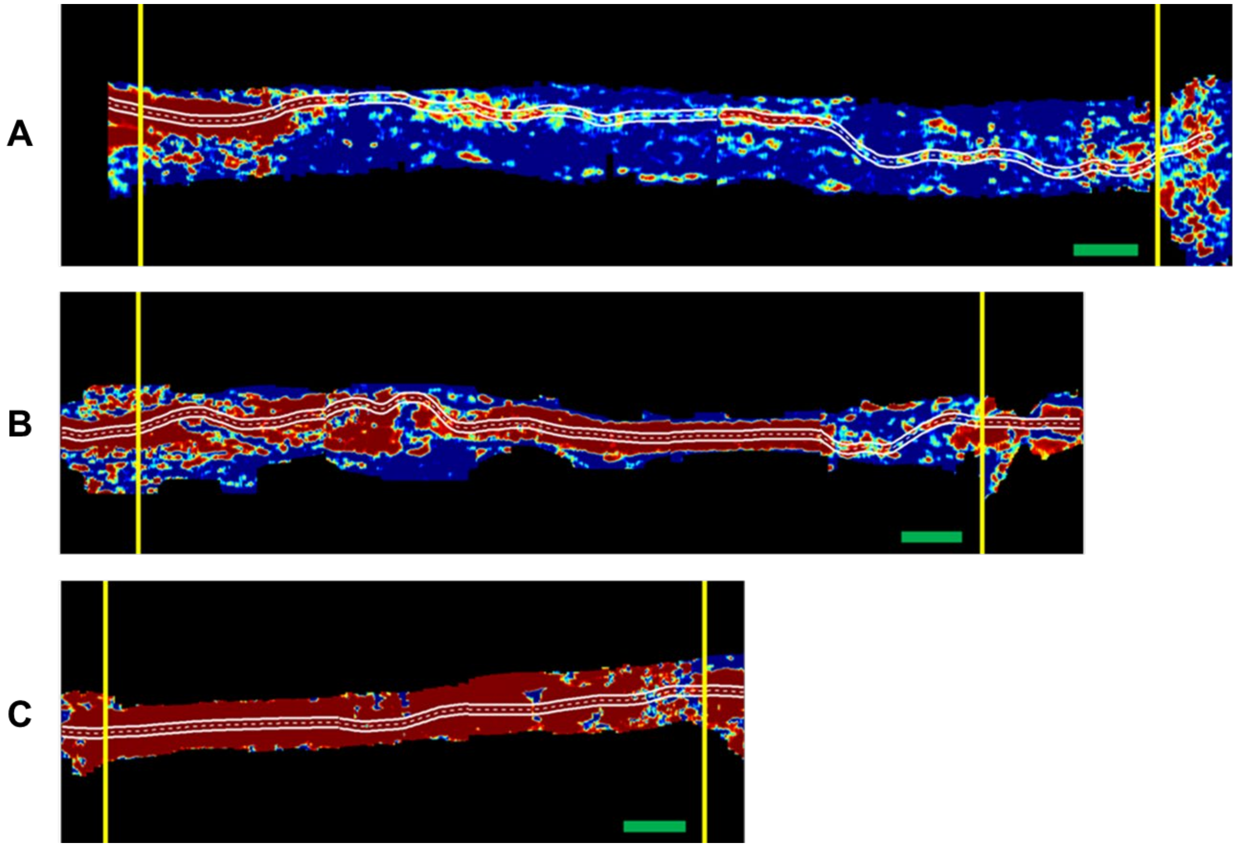

Figure S1. Representative enface phase-retardation images and manually traced optimal nerve connection paths. (A), (B) and (C) are the enface phase retardation images of the whole length of a static, MES, and healthy control sample, respectively. In these images, the color scale from blue to red represents increasing phase retardation within the range of 0.07 to 0.2 deg/μm. The left side corresponds to the proximal end of the nerve. Vertical yellow lines mark the proximal and distal boundaries of the conduit. Dashed white lines indicate the manually traced optimal nerve connection paths, while the solid white lines delineate the 150 μm-wide regions used for averaging. Scale bar = 1mm.

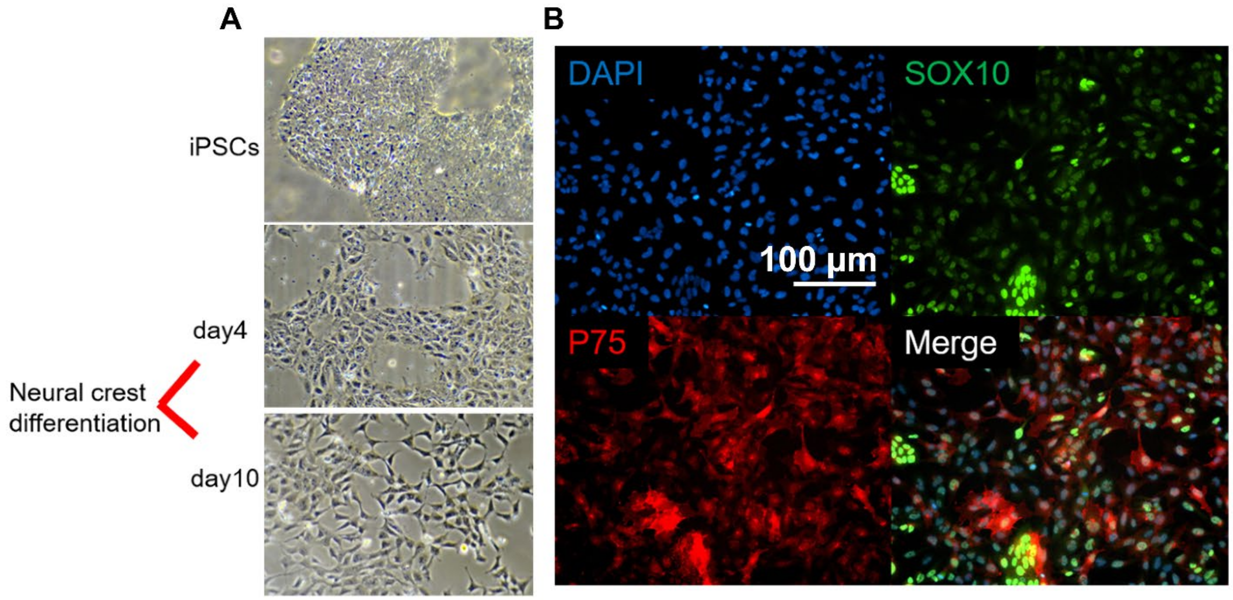

Figure S2. **Derivation and characterization of neural crest stem cells (NCSCs)-like cells from induced pluripotent stem cells (iPSCs).** (A) Bright-field images showing the morphology of iPSCs and NCSC-like cells. (B) Immunofluorescent images of neural crest markers SOX10 and P75NTR, counter-stained with DAPI.

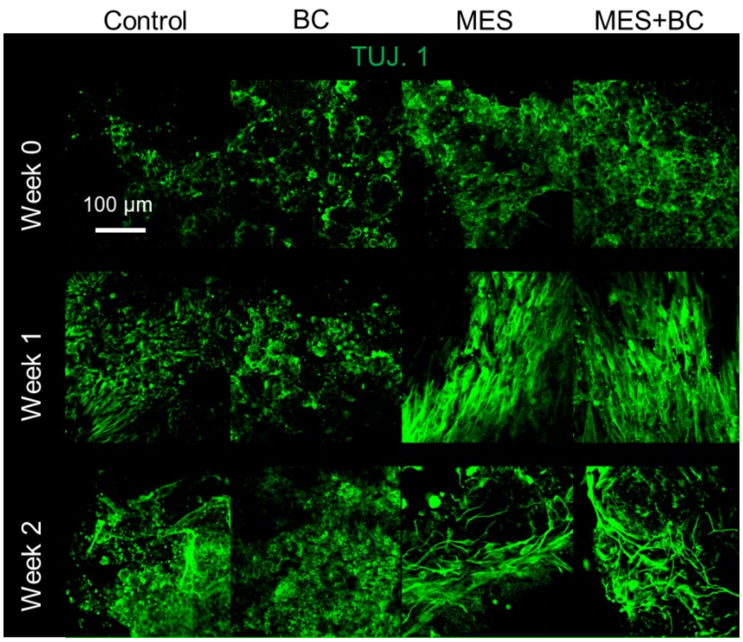

Figure S3. Green channel of fluorescence images (TUJ. 1) in Figure 2B.

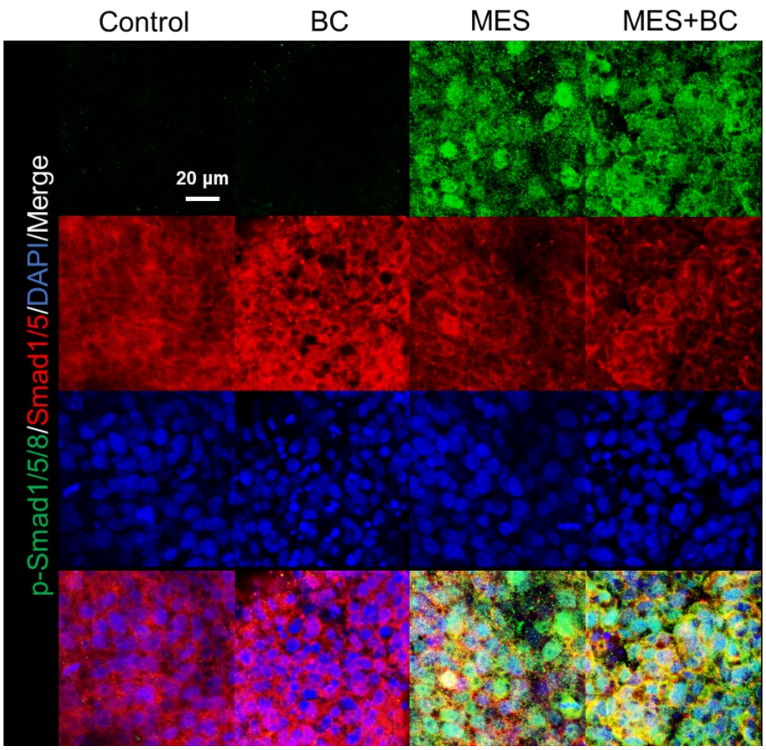

Figure S4. Effect of physical/biochemical stimulation on the expression of Smad. Confocal images showing the expression of p-Smad1/5/8 (green) and Smad1/5 (red) after the first stimulation at week 0 under the Control, Biochemical stimulation (BC), Mechano-electrical stimulation (MES), and MES+BC conditions. The cells were stimulated for 2 hours before sample fixation.

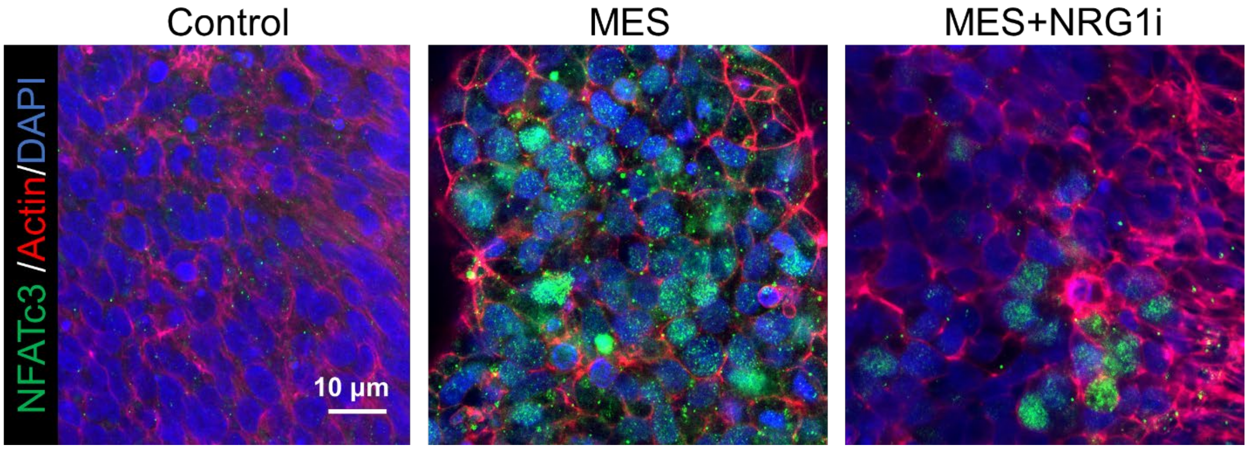

Figure S5. **Effect of NRG1 inhibitor on NFATc3 expression under Control, mechano-electrical stimulation (MES), and MES+NRG1 inhibitor (MES+NRG1i) conditions.**

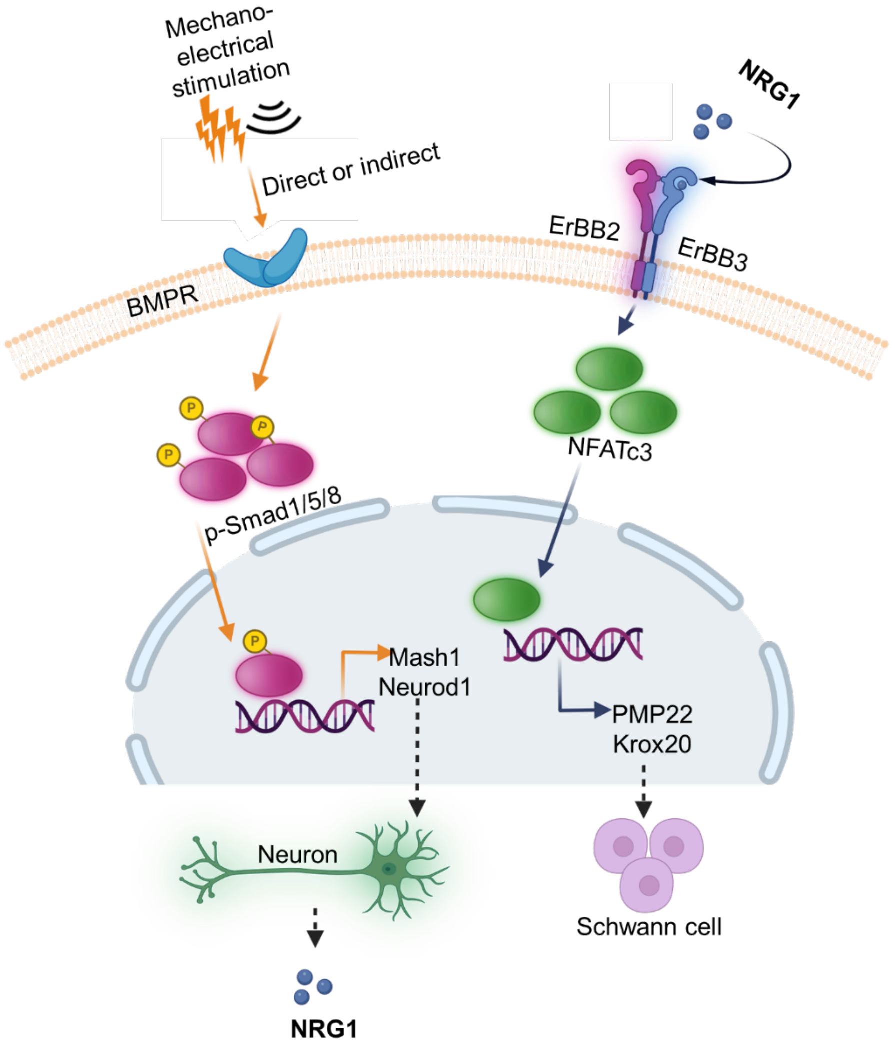

Figure S6. Schematic of the signaling mechanism that mediates the MES-induced NCSC-like cells differentiation towards neurons and Schwann cells.

Supplemental table

Table S1 (refer to Figures 2-4). Human primers for the qRT-PCR analysis.

Primer	Forward	Reverse
GAPDH	5’-ATGGGGAAGGTGAAGGTCG-3’	5’-TAAAAGCAGCCCTGGTGACC-3’
NEUROD1	5’-AAAGCCCTCTGACTGATTGCA-3’	5’-GGACGGTTCGTGTTTGAAAGA-3’
MASH1	5’-ACTTGAACTCCATGGCCGGCT-3’	5’-CCAGTTGGTGAAGTCGAGAAG-3’
NGN2	5’-CATCAAGAAGACCCGTAGACTGA-3’	5’-TCTCGATCTTGGTGAGCTTGG-3’
KROX20	5’-TGCACCTAGAAACCAGACCTT-3’	5’-ATGCCTGCACTCACAATATTG-3’
NCAM1	5’-TGGAATGCTGAGTATGAGGTC-3’	5’-TGAACACAAAATGAGCCGCCT-3’
PMP22	5’-TGTAGCACCTCTTCCTCAG-3’	5’-GAGTTGGCAGAAGAACAGGAAC-3’

Supplemental methods

Synthesis of electrospun poly(vinylidenefluoride-trifluoroethylene)(P(VDF-TrFE)) scaffolds

P(VDF-TrFE) fibers having an average fiber diameter of 500 nm were synthesized using an electrospinning technique as described previously [S1]. Briefly, a solution of 7 wt.% P(VDF-TrFE) (70:30 mol%, Solvay) dissolved in a solvent mixture of N,N-dimethylformamide (DMF) (Sigma) and acetone (Fisher) in a 60:40 volume ratio, supplemented with 1.5 wt.% pyridinium formate (PF) buffer (Sigma), was prepared. Electrospinning was performed under optimized conditions, including a needle tip-to-collector distance of ~10 cm, an applied voltage of −15 to −20 kV, a solution feed rate of 6 mL/h, and an absolute humidity of 7.6 g m^{-3} at room temperature (23 °C). A rotating wheel with an angular velocity of 47.9 m s^{-1} was used for fiber collection, resulting in aligned fibrous scaffolds. The process duration was adjusted to produce scaffolds with a thickness of approximately 200 μm. To enhance piezoelectric properties, the scaffolds were annealed at 90°C for 24 hours [S1].

Surgical procedures and nerve-conduit harvesting

Animal experiments were conducted as previously described [S2] and approved by the University of California-Riverside (UCR) Institutional Animal Care and Use Committee (IACUC, protocol 20210016), in compliance with ethical regulations. Adult Sprague Dawley rats (n = 9, Taconic) weighing ~300 g were randomly divided into three groups. Rats underwent sciatic nerve transection surgery, and the severed nerve ends were sutured to P(VDF-TrFE) conduits, forming a 15 mm nerve gap. Experimental rats (MES group, n = 3) received periodic shockwave treatments (twice a week) post-surgery, while control rats (Static group, n = 3) did not receive mechano-electrical stimulation. No sciatic nerve transection or conduit transplantation was performed on Healthy control rats (n = 3). To apply the mechano-electrical stimulation, a therapeutic shockwave system was used to activate the piezoelectric P(VDF-TrFE) conduits bridging the transected nerves, generating 3 Hz of pulsed biphasic electric signals with 200 $mV_{p\text{-}p}$. At 12 weeks post-surgery, rats were euthanized. Implanted conduits were excised and subjected to further analyses. All samples were fixed in 2% glutaraldehyde/2% paraformaldehyde solution at 4 °C for 48 hours before OCT imaging and histological analysis.

Polarization-sensitive optical coherence tomography (PS-OCT) imaging

Fixed samples were imaged with a custom-built spectral domain polarization-sensitive optical coherence tomography (PS-OCT) system with a central wavelength of 1310 nm and a full-width at half-maximum (FWHM) bandwidth of 68 nm. The axial and lateral resolutions of the system are 11 μm and 37 μm, respectively, and an imaging depth of 2 mm. A comprehensive description of the hardware, scanning, and data processing pipeline has been reported previously [S2]. In brief, each sample was scanned volumetrically in a series of overlapping sections to capture the full length, with each section covering a 4.5 mm × 4.5 mm lateral field of view and an overlap of approximately 1 mm between adjacent sections to ensure proper registration and alignment. The sections were manually aligned when generating the full length of the sample. From volumetric data, structural intensity images were generated following the standard Fourier domain processing method [S3]. The surface of the nerve within the conduit was manually delineated in intensity images and served as the polarization reference for computing cumulative phase-retardation images with a Stokes vector approach [S4]. The slope of the rising portion of the cumulative phase retardation curve, which is proportional to birefringence [S5], was measured using a linear least square fit to generate enface phase retardation image.

For 3D visualization, structural intensity images were compiled in Amira 3D visualizing software. In the rendered 3D images, the conduit structure was displayed in grayscale, while the nerve was visualized in color (**Figure 1A-C**). To enhance the visibility of the nerve within the conduit, the conduit intensity was rendered semi-transparent.

From the enface phase retardation image, the optimal nerve connection path was manually traced (**Figure S1**). Phase retardation along this path was measured by averaging the phase retardation values within a 150 μm diameter window centered on the traced path. For each rat, the mean phase retardation values were calculated over 1 mm long segments at the proximal and distal ends inside the conduit. The mean

and standard error of these measurements were used to quantitatively compare the three experimental groups (**Figure 1D**).

Histology

After PS-OCT imaging, the harvested tissues were sectioned using a cryostat (Leica) [S2]. Longitudinal nerve-conduit sections were immunohistochemically labeled with anti-SOX2 (Proteintech, 11064-1-AP), anti-p75NTR (Cell Signaling, 8238T), or anti-NRG1 (Proteintech, 10527-1-AP) antibodies, followed by secondary antibodies conjugated to horseradish peroxidase (Jackson ImmunoResearch) and DAB substrate (Fisher). Samples labeled with anti-p75NTR and anti-SOX2 were counterstained with hematoxylin to visualize cell nuclei and imaged using a bright-field microscope (Olympus). Quantification of nuclear SOX2 intensity, cytoplasmic P75-NTR, and NRG1 over the area of imaging fields was conducted using ImageJ software.

Cell culture

All experiments involving human stem cells were approved by the UC Riverside Institutional Review Board (IRB; HS11-124) and Stem Cell Research Oversight Committee (SCRO; SC20210002). Induced pluripotent stem cells (iPSCs) were derived from BJ-2522 human neonatal foreskin fibroblast cells (ATCC) and their pluripotency was confirmed [S6]. The normal karyotype of the iPSC line was confirmed. Cells were tested for mycoplasma contamination using a luminescence-based method with a Lonza Lucetta Luminometer authenticated by the Stem Cell Center in University of California, Riverside. There was no bacteria or fungus contamination observed under either the bright field microscope or the fluorescence microscope. Neural crest stem cells (NCSCs) were derived from iPSCs using a protocol described elsewhere [S7]. Briefly, passage-43 iPSCs were thawed in 37 °C bath and maintained in mTeSR1 (Stemcell technologies) media on Geltrex-coated 6-well plate (Falcon). Once confluent, cells were detached using Accutase (Fisher) and seeded onto electrospun P(VDF-TrFE) scaffolds at a density of 150,000 cells/scaffold (7 mm x 5 mm). The modified Dalton NCSC differentiation protocol was used to guide the differentiation of the seeded iPSCs into trunk NCSCs [S7, S8]. NCSC-like cells were then subjected to physical stimulation (MES), using electrospun P(VDF-TrFE) scaffolds activated by the hydroacoustic actuator as previously described [S1]. Biphasic electric pulses at 3 Hz with an amplitude of 200 mV_{p-p} were generated on the surface of P(VDF-TrFE) scaffolds under the hydroacoustic actuation, directly stimulating the cells. Biochemical stimulation (BC; NRG1, 10 ng/mL) or the combination of both (MES+BC) were also separately applied. Cells cultured on scaffolds without MES or BC were used as the Control group. The cells were stimulated for 2 hours for various overall culture durations including 0 week (cells were stimulated only once), 1 week, 2 weeks, 3 weeks, or 4 weeks before fixation in 4% PFA (Fisher) or lysed using RLT buffer (Qiagen) for imaging and gene expression analyses, respectively. For mechanistic studies, signaling inhibitors including Smad inhibitor (Dorsomorphin dihydrochloride, 5 μM, Tocris) and NRG1 inhibitor (Seribantumab, 5 μM, MedChemExpress) were used. These inhibitors were individually applied under the MES condition for a week before cell fixation or lysing.

Immunofluorescence imaging

Fixed cells were immune-stained with various markers including, anti-TUJ.1 (Fisher, MA1118), anti-GALC (Proteintech, 11991-1-AP), anti-NEUN (Abcam, ab177487), anti-NRG1, anti-smad1/5 (Santa Cruz Biotechnology, sc-7965, sc-101151), anti-p-Smad1/5/8 (Sigma, AB3848-I), and anti-NFATc3 (DSHB, PCRP-NFATC3-2A12), followed by appropriate secondary antibodies conjugated with fluorophores (Goat Anti-Mouse IgG (H+L) with Alexa Fluro@ 488 or 594 and Goat Anti-Rabbit IgG (H+L) with Alexa Fluro@ 488 or 594, Jackson ImmunoResearch). A confocal microscope (Zeiss) was used to image the expression of different markers. ImageJ software was used to quantify fluorescence intensity or protein nuclear localization. For the quantification of nuclear localization, the Coloc2 plugin in ImageJ was used and the resulting Pearson's coefficient value was used to describe the degree of NFATc3 nuclear localization. A value of +1 indicates complete nuclear localization, while -1 indicates complete cytoplasmic localization [S9-S11].

Gene expression analysis

Total RNA was extracted using an RNeasy Micro Kit (Qiagen) followed by cDNA synthesis using an iScript cDNA Synthesis Kit (Bio-rad). RT-qPCR was performed to determine the phenotypic expression of neuronal, astrocytic, and oligodendrocytic genes (**Table S1**). Raw data were analyzed by the comparative threshold cycle (C_T) method using the expression of Gapdh as an endogenous control.

Elisa assay

Elisa (Enzyme-linked immunosorbent assay) was used to assess extracellular NRG1 secretion. At 2 weeks under various cell culture conditions such as Control, BC, MES, and MES+BC conditions, supernatant was collected 0, 2, 4, 8, and 24 hours after the last daily stimulation. Elisa was performed following the manufacture protocol (R&D Systems).

Statistical analysis

All experiments were conducted with a minimum of triplicate biological samples and data are presented as mean ± standard error of means. Comparison of experimental groups for statistical significance was determined using the IBM SPSS software with either one-way ANOVA with Tukey's HSD post-hoc test or a two-sample student *T*-test. Statistical significance was reported when a '*p*' value was less than 0.05 and 0.01.

Supplemental references

S1. Tai, Y., Ico, G., Low, K., Liu, J., Jariwala, T., Garcia-Viramontes, D., Lee, K.H., Myung, N.V., Park, B.H., and Nam, J. (2021). Formation of 3D Self-Organized Neuron-Glial Interface Derived from Neural Stem Cells via Mechano-Electrical Stimulation. Adv Healthc Mater *10*, e2100806. 10.1002/adhm.202100806.

S2. Tai, Y., Tonmoy, T.I., Win, S., Brinkley, N.T., Park, B.H., and Nam, J. (2023). Enhanced peripheral nerve regeneration by mechano-electrical stimulation. NPJ Regen Med *8*, 57. 10.1038/s41536-023-00334-y.

S3. Mitsui, T. (1999). Dynamic range of optical reflectometry with spectral interferometry. Jpn J Appl Phys 1 38, 6133-6137. Doi 10.1143/Jjap.38.6133.

S4. Park, B.H., Saxer, C., Srinivas, S.M., Nelson, J.S., and de Boer, J.F. (2001). In vivo burn depth determination by high-speed fiber-based polarization sensitive optical coherence tomography. J Biomed Opt 6, 474-479. Doi 10.1117/1.1413208.

S5. Hee, M.R., Huang, D., Swanson, E.A., and Fujimoto, J.G. (1992). Polarization-Sensitive Low-Coherence Reflectometer for Birefringence Characterization and Ranging. J Opt Soc Am B 9, 903-908. Doi 10.1364/Josab.9.000903.

S6. Torrez, L.B., Perez, Y., Yang, J., Zur Nieden, N.I., Klassen, H., and Liew, C.G. (2012). Derivation of neural progenitors and retinal pigment epithelium from common marmoset and human pluripotent stem cells. Stem Cells Int 2012, 417865. 10.1155/2012/417865.

S7. Huang, M., Miller, M.L., McHenry, L.K., Zheng, T., Zhen, Q., Ilkhanizadeh, S., Conklin, B.R., Bronner, M.E., and Weiss, W.A. (2016). Generating trunk neural crest from human pluripotent stem cells. Sci Rep 6, 19727. 10.1038/srep19727.

S8. Menendez, L., Kulik, M.J., Page, A.T., Park, S.S., Lauderdale, J.D., Cunningham, M.L., and Dalton, S. (2013). Directed differentiation of human pluripotent cells to neural crest stem cells. Nat Protoc 8, 203-212. 10.1038/nprot.2012.156.

S9. Costes, S.V., Daelemans, D., Cho, E.H., Dobbin, Z., Pavlakis, G., and Lockett, S. (2004). Automatic and quantitative measurement of protein-protein colocalization in live cells. Biophys J 86, 3993-4003. 10.1529/biophysj.103.038422.

S10. Dunn, K.W., Kamocka, M.M., and McDonald, J.H. (2011). A practical guide to evaluating colocalization in biological microscopy. Am J Physiol Cell Physiol 300, C723-742. 10.1152/ajpcell.00462.2010.

S11. Manders, E.M.M., Verbeek, F.J., and Aten, J.A. (1993). Measurement of co-localization of objects in dual-colour confocal images. J Microsc 169, 375-382. 10.1111/j.1365-2818.1993.tb03313.x.
